# Pan-cancer analysis of cholesterol metabolism reveals the uptake as a modulator of tumor immune features and of the KRAS pathway

**DOI:** 10.1007/s13402-026-01171-z

**Published:** 2026-02-12

**Authors:** Ana Luísa Machado, Ana Pinto, Joana Carvalho, Verónica Fernandes, Luísa Pereira, Jessica Roelands, Jorge Gonçalves, N. F. C. C. Miranda, Maria José Oliveira, Sérgia Velho

**Affiliations:** 1 i3S – Instituto de Investigação e Inovação em Saúde, Universidade do Porto, Rua Alfredo Allen nº208, Porto, 4200-135 Portugal; 2 FMUP – Faculdade de Medicina, Departamento de Patologia, Universidade do Porto, Alameda Prof. Hernâni Monteiro, Porto, 4200-319 Portugal; 3 ESS – Escola Superior de Saúde, Instituto Politécnico do Porto, Rua Dr. António Bernardino de Almeida nº400, Porto, 4200-072 Portugal; 4 IPATIMUP – Instituto de Patologia e Imunologia Molecular da Universidade do Porto, Rua Júlio Amaral de Carvalho nº45, Porto, 4200-135 Portugal; 5 Department of Pathology, Leiden University Medical Center, Albinusdreef 2, Leiden, ZG 2333 the Netherlands; 6 UCIBIO, i4HB, Faculdade de Farmácia, Universidade do Porto, Rua de Jorge de Viterbo Ferreira nº228, Porto, 4050-313 Portugal; 7 INEB – Instituto Nacional de Engenharia Biomédica, Universidade do Porto, Rua Alfredo Allen nº208, Porto, 4200-135 Portugal; 8 ICBAS - Instituto de Ciências Biomédicas Abel Salazar, Universidade do Porto, Rua de Jorge de Viterbo Ferreira nº228, Porto, 4050-313 Portugal

**Keywords:** Cancer, Cholesterol metabolism, Tumor microenvironment, KRAS

## Abstract

**Purpose:**

Cholesterol dysregulation plays a central role in tumor progression and has emerged as a potential therapeutic target. Its dysregulation within the tumor microenvironment is increasingly considered a strong modifier of multiple cancer traits. Targeting cholesterol, particularly its biosynthetic pathway, has been explored to enhance therapy, yet outcomes remain inconsistent, likely reflecting a tumor-specific reprogramming of cholesterol metabolism.

**Methods:**

TCGA and GTEx transcriptomic data from 11,735 samples was analyzed to conduct an integrative assessment of key cholesterol-related processes—biosynthesis, uptake, storage, efflux, and catabolism—across 26 cancer types.

**Results:**

Cholesterol metabolism dysregulation was highly heterogeneous among tumors, affecting different metabolic pathways, the magnitude of those differences and the direction relative to normal tissue. Notably, we identified cholesterol uptake as the most consistently altered pathway across tumors, positively correlated with tumor aggressiveness and poorer patient survival. Uptake correlated positively with inflammatory pathways (e.g., *Complement System* and *IL-6-JAK-STAT3 Signaling*) and with immune microenvironment features, including Tregs, cytotoxic and CD4^+^ T cells, eosinophils, endothelial cells, as well as elevated expression of immune checkpoints. Cholesterol uptake also correlated with enhanced KRAS signaling. Particularly, *KRAS* mutations were more frequent in tumors with high uptake scores, and those patients had the worst overall survival. Nonetheless, high uptake tumors lacking *KRAS* mutations had higher KRAS signaling scores than *KRAS*-mutant tumors with low uptake, highlighting a central role of cholesterol uptake on the regulation of KRAS signaling.

**Conclusion:**

In summary, cholesterol uptake emerges as a conserved driver of tumor aggressiveness and a promising therapeutic target to synergize with immunotherapy and KRAS inhibition.

**Graphical abstract:**

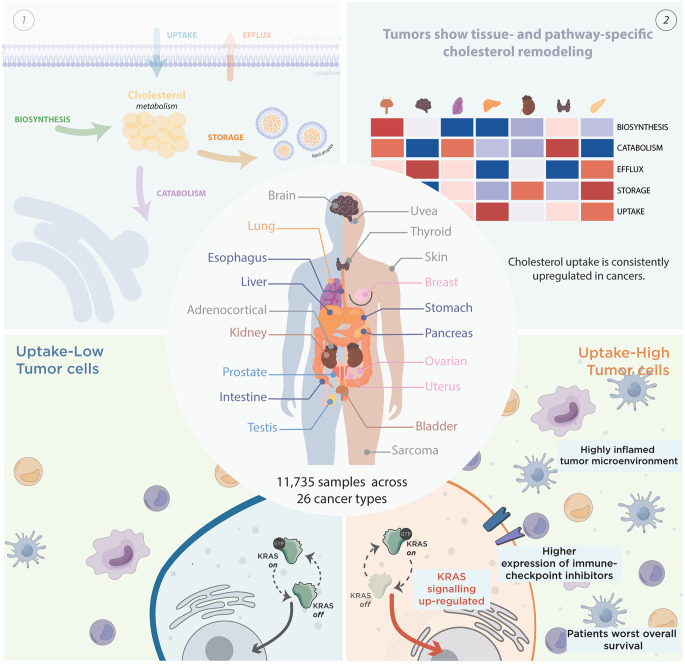

**Supplementary Information:**

The online version contains supplementary material available at 10.1007/s13402-026-01171-z.

## Introduction

Cholesterol is an essential molecule, as it forms part of cell membranes and is involved in the normal function of the body by producing hormones and vitamin D [1]. During malignant transition, cell metabolism is reprogrammed, with cancer cells showing increased capacity to synthesize, internalize, and accumulate cholesterol [1].

Following its reprogramming, cholesterol contributes to various cancer cell processes. For instance, cancer cells boost proliferation by exploiting cholesterol metabolism for membrane synthesis and energy [1]. Moreover, low- or high-density lipoproteins (LDL or HDL) exposure shifts colorectal cancer metabolism to glycolysis, supporting rapid proliferation [2]. Additionally, cholesterol affects drug permeation by altering membrane dynamics. Notably, treatment of colorectal cancer cells with oxaliplatin decreased membrane fluidity, further restored in resistant cells, concomitant with increased membrane cholesterol content [3]. Accordingly, combining oxaliplatin with cholesterol biosynthesis inhibition increases *KRAS*-mutated colorectal cancer cell lines sensitivity both in vitro and in vivo [4, 5]. In addition, cholesterol contributes to the stability and rigidity of lipid rafts, a platform for signaling receptors and adhesion molecules. Consequently, cholesterol profoundly impacts cancer signaling, migration, and invasive potential of breast, colorectal, prostate, gastric, and other tumors [6–9].

Beyond its effects on cancer cells, cholesterol plays a pivotal role in creating a pro-tumorigenic immune microenvironment [10–14]. Accordingly, high levels of cholesterol were associated with CD8^+^ T cells dysfunction (through immune-checkpoint upregulation), exhaustion, and exclusion from intraepithelial regions of the tumor [10, 11]. Additionally, cholesterol metabolites were shown to reduce dendritic cell migration to lymph nodes, facilitating immune evasion [14]. Cholesterol-lowering drugs reversed these effects by restoring dendritic cells and T cell functionality, increasing CD8^+^ T cell tumor infiltration, promoting activation of natural killer cells, and decreasing the number of pro-tumorigenic macrophages [12, 13].

Recently, several clinical trials have shown that statin therapy, inhibiting cholesterol biosynthesis, improves cancer patients survival [15]. According to a randomized trial, statins doubled median overall survival of advanced hepatocellular carcinoma patients [16]. However, others reported no evidence of statins improvement on advanced hepatocellular or ovarian cancer patients’ overall survival [17, 18]. Interestingly, studies indicate that cholesterol biosynthesis inhibition effects vary among cancer types, possibly explaining the distinct therapeutic efficacy of statins [19, 20]. Notwithstanding, cholesterol metabolism is a complex process involving multiple interconnected pathways, including biosynthesis, catabolism, efflux, storage, and uptake [1, 21]. As such, these processes should be considered when exploring cholesterol metabolism as a target for cancer therapy.

Here, we leveraged transcriptomic data to perform a novel integrative analysis encompassing the five processes involved in cholesterol metabolism across multiple cancer types. This comprehensive pan-cancer approach provides insights into the context-dependent effects of cholesterol reprogramming in tumors and its potential role as a modulator of cancer cell hallmarks and immune function. Our study highlights cholesterol uptake as a fundamental process that regulates KRAS signaling and the tumor immune microenvironment, impacting patient survival.

## Methods

### Data acquisition and normalization

RNA-sequencing data (STAR counts) from 26 TCGA cancer projects were obtained using TCGAbiolinks R package (v2.31.3). Gene expression read counts from normal tissues of the cancer types with limited or no normal samples available in TCGA (Brain, Pancreas, Ovarian, Testis, and Skin), were supplemented with data obtained from the GTEx Portal. For certain organs, however, no normal tissue data were available in either source. Gene symbols were mapped to official HGNC symbols, and genes without a valid symbol or annotation were excluded. Normalization was performed in two steps: (i) within-lane correction for gene-specific effects (e.g., GC content) and (ii) between-lane correction for sample-related differences (e.g., sequencing depth), using the EDASeq package (v2.40.0). Finally, data were quantile-normalized with preprocessCore (v1.65.0). After normalization, a single tumor and normal sample per patient was retained for analysis and the data was log2-transformed. Normalized and log2- transformed gene expression data from normal and tumor samples were subsequently combined for further analysis. Clinical data was sourced from cBioPortal [22, 23]. Somatic mutation data for the TCGA cohorts were obtained from the TCGA Multi-Center Mutation Calling in Multiple Cancers (MC3) project using the TCGAbiolinks R package. Samples were classified as mutant for a given gene if a somatic mutation was reported in the MAF files. Samples were considered wild-type if they were present in the MAF dataset but no mutation was detected for that gene. Samples lacking mutation data were excluded from the analyses in which mutation status was considered.

### Cholesterol metabolic pathways

**Fig. 1:**
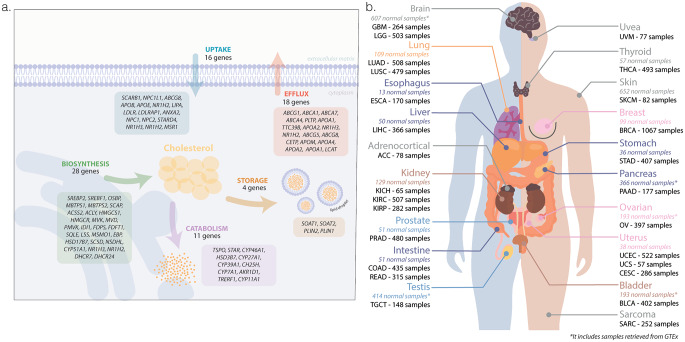
Overview of the pan-cancer and cholesterol metabolism pathways atlas. **a**. Schematic representation of cholesterol metabolism pathways, indicating the genes included in each pathway for ssGSEA-based enrichment scoring; **b**. Anatomical distribution of 26 TCGA cancer projects across 19 tumor sites, with corresponding numbers of tumor and normal samples. ACC, adrenocortical carcinoma; BLCA, bladder carcinoma; BRCA, breast carcinoma; CESC, cervical carcinoma; COAD, colon adenocarcinoma; ESCA, oesophageal carcinoma; GBM, glioblastoma multiforme; KICH, kidney chromophobe carcinoma; KIRC, kidney renal clear cell carcinoma; KIRP, kidney renal papillary carcinoma; LGG, low grade glioma; LIHC, hepatocellular carcinoma; LUAD, lung adenocarcinoma; LUSC, lung squamous cell carcinoma; PAAD, pancreatic carcinoma; PRAD, prostate carcinoma; READ, rectal adenocarcinoma; OV, ovarian serous adenocarcinoma; SARC, sarcoma; SKCM, skin melanoma; STAD, stomach carcinoma; TGCT, testicular germ cell tumor; THCA, thyroid carcinoma; UCEC, uterine carcinoma; UCS, uterine carcinosarcoma; UVM, uveal melanoma

Gene sets reflecting the five specific cholesterol-related pathways studied were manually curated (Fig. 1a). Manual curation was performed using cholesterol-related GO pathways (e.g., biosynthesis, catabolism, efflux, storage, and receptor-mediated transport) as reference (Supplementary Table S1). Only genes specifically related to the cholesterol metabolism processes and associated with upregulation of the pathway were retained. Genes overlapping multiple pathways were assigned to the pathway in which their functional role was most prominent, based on evidence from the literature. When no clear preference could be established, genes were retained in all relevant pathways. The normalized z-scores for gene expression, in normal and tumor samples, were visualized using an unsupervised hierarchical clustering (Ward.D2 linkage) heatmap generated with ComplexHeatmap (v2.22.0). Further, single-sample gene set enrichment analysis (ssGSEA) was applied to the log2-transformed, normalized gene expression matrix using GSVA R package (v2.0.7) [24]. The resulting enrichment scores were standardized across samples to calculate z-scores. Distributions of pathway z-scores across organs of origin, stage, and histological/molecular subtypes were visualized in ggplot2 (v3.5.2), grouping tumor and normal samples by organ of origin. The mean z-scores for normal and tumor samples were calculated for each organ of origin and visualized using spider plots generated with the fmsb package (v0.7.6). Normal and tumor samples were compared using t-tests or Mann-Whitney tests, depending on data distribution assessed with the Shapiro-Wilk test. Comparisons across tumor stage and histological/molecular subtypes were performed using ANOVA or Kruskal-Wallis tests, as appropriate. Multiple test corrections were performed by calculation the FDR using Tukey’s or Dunn’s tests, depending on data distribution.

### Samples stratification by cholesterol pathway scores

For each of the five manually curated cholesterol-related pathways, pan-cancer tumor samples were divided into three groups (low, medium, and high) according to the values of the cholesterol-related pathway scores. The lowest third were assigned to the low group, the middle third to the medium group, and the highest third to the high group.

### Survival analysis

Cox proportional hazards regression models were applied to assess the association between cholesterol-related pathway z-scores and overall survival both within pan-cancer and individual TCGA projects. Pathway z-scores and tumor stage (or histological grade for GBM and LGG projects) were included as covariates. For the combined analysis, samples were first stratified by uptake score into high and low groups, and within each of these, further subdivided according to the other cholesterol-related pathway scores (stratification procedure is described below) or to the *KRAS* mutational status. Cox models were further adjusted for the other pathway z-scores. Hazard ratios with 95% confidence intervals were estimated using the survival (v3.7.0) and broom (v1.0.8) R packages, and forest plots were generated to visualize effect sizes across projects. Predicted survival curves for specific combinations of covariates were generated using the survfit function, and visualized with the survminer R package (v0.5.0).

### Oncogenic gene set enrichment analysis

To assess the enrichment of oncogenic pathways, ssGSEA was applied to log2-transformed, normalized expression data. Gene sets used to define enrichment of specific tumor-related pathways were obtained from multiple sources. From the 50 Hallmark pathways available in MSigDB, we selected 35 that were relevant in a pan-cancer context, excluding those with tumor type-specific relevance. Additionally, we included 7 other pathways related to cancer signaling to provide a broader overview of the principal dysregulated pathways in cancer (e.g. p38 MAPK, EGFR, PTEN, and VEGFA-VEGFR signaling). Finally, we selected 9 gene sets from the MSigDB C5 collection, specifically the GO Biological Process subset, to cover processes related to membrane dynamics, a fundamental process regulated by cholesterol metabolism (e.g. membrane assembly, membrane docking, and membrane biogenesis) and ferroptosis, a metabolic form of regulated cell death. The complete list of gene sets used is provided in Supplementary Table S2.

### Immune cell deconvolution and ESTIMATE

To estimate the relative abundances of specific immune cell subsets in tumor samples, we employed the Consensus Tumor Microenvironment Cell Estimation (ConsensusTME) algorithm. The algorithm uses integrated curated and validated gene sets from multiple sources on a per- cancer-type basis to allow a deconvolution of immune cells through bulk transcriptomics data. We applied the R package ConsensusTME (v0.0.1.9000) specifying the corresponding tumor type and using ‘ssgsea’ as the statistical method. To assess overall stromal and immune cell infiltration within the tumor, we applied the ESTIMATE algorithm (v1.0.13) to the log2-transformed, normalized gene expression matrix in R sofware (v4.4.2).

### Correlation matrices

Pearson correlation was used to evaluate associations between the studied cholesterol-related pathways and distinct oncogenic pathways, as well as tumor microenvironment composition (ESTIMATE and ConsensusTME scores), both in a pan-cancer setting and within each TCGA project individually. The resulting correlation was visualized using the ComplexHeatmap package or as scatter plots with ggplot2. Pearson correlation was additionally applied to examine the relationship between cholesterol uptake and *KRAS* mutation in all the stratified correlation analyses. These correlation analyses were visualized in a scatter plot with fitted simple linear regression lines, generated with ggplot2.

### Differential gene expression (DEG) and functional enrichment analysis

Differentially expressed genes were identified through pairwise comparison between the extreme uptake groups (low versus high; with or without stratification by *KRAS* mutational status), and between low and high catabolism groups, using limma package (v. 3.62.2). A standard linear model was fitted, followed by an empirical Bayes procedure to stabilize variance estimates. A moderated t-statistic, derived from the Bayesian approach, was used as the primary test statistic, followed by multiple test correction through the Benjamini-Hochberg method to estimate the false discovery rate. All analyses were performed using the limma package with default settings. Further, for functional enrichment analysis, gene set enrichment analysis (GSEA) was performed using gseGO function in the clusterProfiler R package (v.4.14.6). Genes were ranked by log fold-change (logFC) values, and analyses were conducted for Gene Ontology biological process (BP) categories. To explore relationships among enriched gene sets, the emapplot function from the enrichplot R package (v1.26.6) [25] was used. This function generates network-like plots where nodes represent gene sets and edges reflect gene overlap between sets.

## Results

### Cholesterol metabolism reprograming is highly heterogeneous across tumor types

Cholesterol metabolism is a complex process involving a dynamic balance between biosynthesis, uptake, efflux, storage, and catabolism [25]. To study this multifactorial process, gene sets of the five pathways involved in cholesterol metabolism were manually curated (Fig. 1a). RNA-seq data from a total of 8832 patients across 19 distinct solid cancer types (Fig. 1b) were obtained from TCGA, and compared with 2249 normal tissue RNA-Seq profiles from GTEx and TCGA. The expression of genes defining the cholesterol-related pathways showed a clear pattern across distinct normal tissues. In contrast, tumor samples showed greater heterogeneity, occasionally clustering distinct cancer types together (Supplementary Fig. S1).

To assess the validity of the new gene sets in an organ-specific context, we compared the cholesterol pathway scores across normal tissues. Among analyzed tissues, the liver ranked highest for all cholesterol-related pathways, except for storage, in which the breast ranked highest (Supplementary Fig. S2). However, in tumor samples, although the liver maintained the highest scores, tissue ranking changed markedly (Supplementary Fig. S2).

**Fig. 2:**
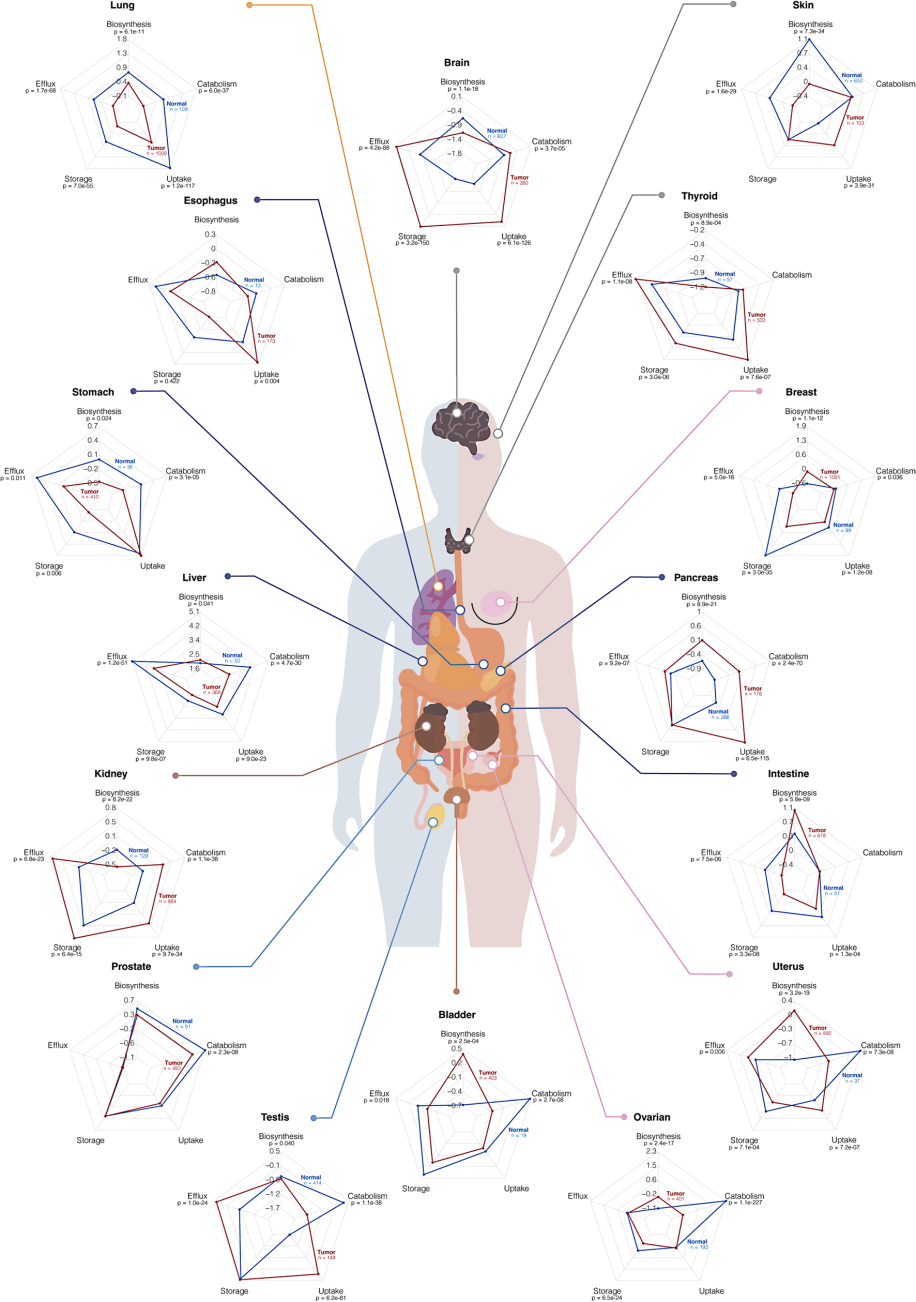
Mean scores comparison of normal and tumor samples cholesterol-related pathways across each organ of origin. *n* refers to the number of samples. *p* denotes the two-sided *p*-value obtained from either a Student’s *t*-test or a Mann–Whitney *U* test, depending on data distribution, which was assessed using the Shapiro–Wilk test. Information on adjacent normal tissue was not available for uvea and adrenocortical samples

Due to organ-specific differences in baseline cholesterol metabolism, cholesterol-related scores were compared between tumors and normal tissue by organ (Fig. 2). This comparison showed a similar number of tumors with decreased or increased cholesterol biosynthesis- and efflux- gene expression scores across organs. In contrast, catabolism and storage scores were predominantly downregulated in tumors (80% and 73% respectively) (Supplementary Table S3). Conversely, genes related to the cholesterol uptake were significantly upregulated in about 67% of tumors (Supplementary Table S3). Of note, the opposite trends observed for storage and catabolism were restricted to neuroendocrine tumors (kidney, brain, and thyroid) in storage, and to kidney, brain, and pancreatic tumors in catabolism (pancreas: no differences in storage; thyroid: non-significant increase of catabolism) (Fig. 2). Moreover, tumors with decreased uptake-related gene expression, contrary to the general increasing, included the lung, liver, intestine, and breast.

### Cholesterol uptake increase is consistently conserved in tumor samples across cancer types

To determine whether the up- and downregulation of cholesterol-related pathways observed in tumors versus normal tissue was consistent across samples, we evaluated the variability of each cholesterol-related pathway. To accomplish this, the pan-cancer coefficient of variation (SD/mean) and the median of cholesterol-related scores were calculated and tumoral and normal values were compared (Supplementary Fig. S3a). Cholesterol uptake had the highest median increase between normal and tumor samples and displayed the lowest heterogeneity across tumor samples, given by its lowest coefficient of variation. Additionally, consistent with previous observations, median catabolism and storage scores tended to decrease, while biosynthesis and efflux scores tended to increase from normal to tumor samples (Supplementary Fig. S3a). Furthermore, compared with normal, tumor samples exhibited higher coefficients of variation for all pathways, indicating greater heterogeneity in cholesterol metabolism among tumors (Supplementary Fig. S3a). Coefficient of variation versus median score plot, across cholesterol-related pathways, and the tumor-specific coefficients of variation histogram, across distinct TCGA projects, are available in the Supplementary Fig. S3b and c.

### Cholesterol metabolism varies across disease stages and subtypes

Next, we compared cholesterol-related pathways scores across disease stages, histological and molecular subtypes to explain the heterogeneity found in tumors and gain insights into how cholesterol-related pathways impact or are impacted along tumor progression.

Comparison of cholesterol-related pathways across tumor stages revealed an inconsistent pattern across all tumor types (Supplementary Fig. S4). In lung, uveal, esophagus, testis, and ovarian cancers, no alterations were found across disease stages. In thyroid cancers, all pathways were altered across disease stages. In the remaining tumors, one or more pathways were altered. Nevertheless, despite some pathways showing significant differences according to disease stage in specific tumors, these changes rarely followed a coordinated progression along stages. This indicates that regulation of cholesterol metabolic pathways is not uniform during disease progression and remains highly influenced by the organ of origin.

In addition, cholesterol-related pathways exhibited substantial differences between histological/molecular subtypes in certain tumors (kidney, breast, stomach, and brain) highlighting subtype-specific heterogeneity (Supplementary Fig. S5 and S6).

### Cholesterol impacts patient survival in a pathway-specific manner

**Fig. 3:**
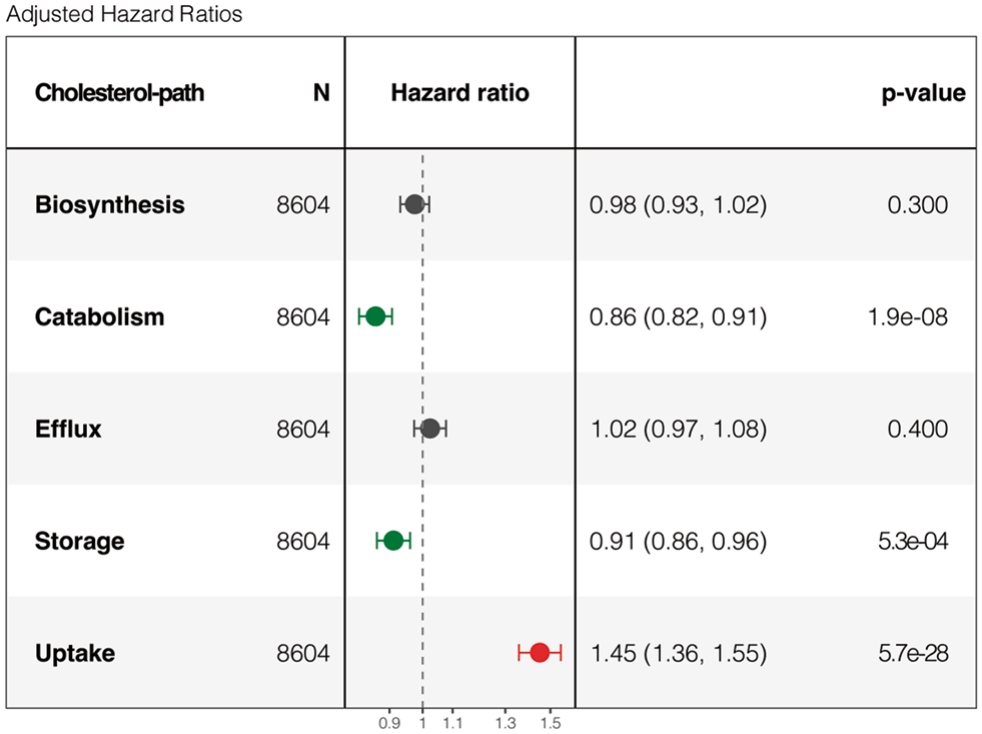
Pan-cancer prognostic significance of cholesterol pathways expression levels. Multivariable Cox proportional hazards models were used to estimate Pan-Cancer hazard ratios and 95% confidence intervals (CI for each cholesterol metabolism pathways). Models were adjusted for tumor stage or grade (GBM and LGG) and for the remaining cholesterol pathways. Tumor-specific hazard ratios are available in Supplementary Fig. S7

Further, pan-cancer association between cholesterol-related pathways and survival was assessed using multivariable Cox proportional hazards models to estimate hazard ratios (HRs), adjusting for stage or grade in the case of glioblastoma (GBM), and low-grade glioma (LGG) (Fig. 3). Higher expression of cholesterol catabolism- and storage-related genes tended to be protective (HR < 1), whereas higher expression of uptake-related genes was associated with increased risk of death (HR > 1). No significant association was observed for biosynthesis- or efflux-related gene expression (Fig. 3). Organ-stratification revealed that catabolism was protective in all tumors showing significant associations, consistent with pan-cancer analysis, including uveal melanoma (UVM), prostate (PRAD), liver (LIHC), kidney chromophobe (KICH), breast (BRCA), and adrenocortical (ACC) tumors (Supplementary Fig. S7b). In contrast to the pan-cancer analysis, stratified analysis for cholesterol storage showed that significant HRs in stomach (STAD) and lung adenocarcinoma (LUAD) were associated with increased risk of death (Supplementary Fig. S7d). Finally, cholesterol uptake HRs across tumor types largely agreed with the pan-cancer analysis, with HRs of ovarian (OV), LGG, GBM, KICH, renal papillary carcinoma (KIRP), and bladder (BLCA) tumors showing higher death risk associated with increased uptake-related gene expression (Supplementary Fig. S7e).

### Cholesterol-related pathways are differentially associated with tumor and immune microenvironment features

**Fig. 4:**
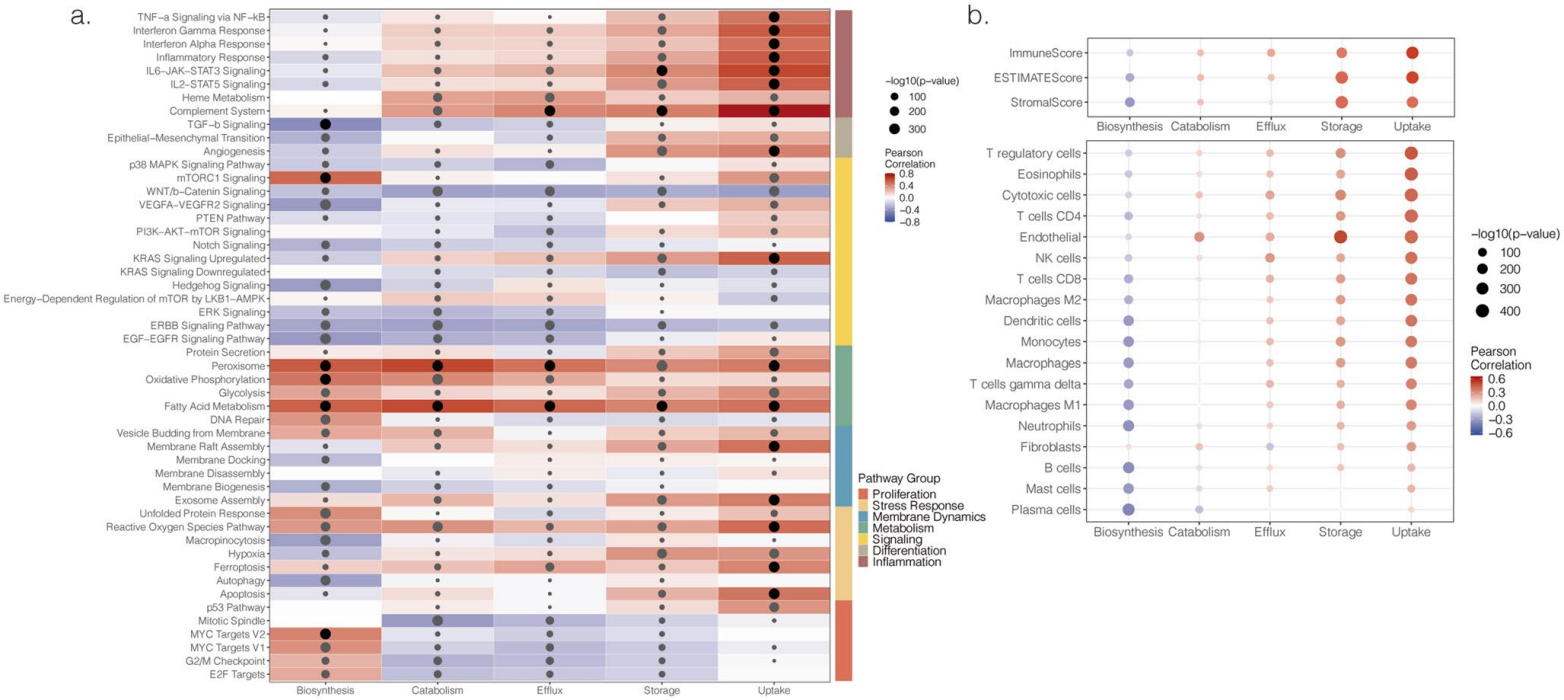
Enrichment of tumor-associated pathways and deconvolution of immune cells population. **a**. Pan-cancer Pearson coefficient correlation between cholesterol metabolism pathways scores and enrichment scores of tumor-associated pathways. Tumor-associated pathways were grouped into seven functional categories; **b**. Pan-cancer Pearson correlation coefficients between enrichment scores of cell-specific immune signatures, obtained using ESTIMATE (top) and ConsensusTME (bottom), and cholesterol metabolism pathways scores. Cells are sorted according to the strength of their Pearson correlation with uptake, in descending order. Significant correlations (*p* < 0.05) are indicated by dots; dot size increases with statistical significance. Correlation matrices are available in Supplementary Fig. S8; tumor-specific correlations are available in Supplementary Fig. S9 and S10

To assess associations between cholesterol-related pathway and tumor features, ssGSEA was used to quantify enrichment of key tumor-associated pathways (e.g. cell cycle regulation, stress response, membrane dynamics, metabolism, signaling, differentiation, and inflammation), and Pearson correlations were calculated between their enrichment scores and cholesterol-related pathway scores (Fig. 4a and Supplementary Fig. S8a).

Uptake showed the highest Pearson correlation coefficients, primarily with tumor-associated pathways involved in inflammation, such as *Complement System* (*r* = 0.75) and *IL-6-JAK-STAT3 Signaling* (*r* = 0.63), and with metabolic pathways, including *Peroxissome* (*r* = 0.44), and *Fatty Acid Metabolism* (*r* = 0.47), and stress response pathways, such as *the Reactive Oxygen Species pathway* (*r* = 0.49) and *Apoptosis* (*r* = 0.45) (Fig. 4a). Likewise, one of the highest correlation coefficients related to uptake was *Kras signaling Upregulated* (*r* = 0.53), one of the most frequently mutated genes across tumors. Catabolism, storage, and efflux displayed correlation patterns resembling those of uptake, though the correlation coefficients were consistently lower (Fig. 4a). Biosynthesis showed a markedly different correlation pattern, displaying strong positive correlations with metabolism and proliferation pathways, and negative correlations with *TGF-b signaling* (*r* = -0.44), *VEGFA-VEGFR2 Signaling* (*r* = -0.38), and *EGF-EGFR Signaling* (*r* = -0.38) (Fig. 4a). Correlation patterns between tumor-associated and our manually curated cholesterol-related set were largely conserved across tumor types (Supplementary Fig. S9), thus reinforcing cholesterol as a regulator of the identified pathways.

To further evaluate the relevance of the five cholesterol-related pathways in the tumor immune microenvironment, ESTIMATE was used to calculate immune scores, and Consensus TME predicted relative leukocyte proportions subpopulations per sample. Pearson correlations were then calculated between these algorithm scores and cholesterol-related pathway scores (Fig. 4b and Supplementary Fig. S8b). Cholesterol uptake showed a prominent positive correlation with the immune score, predominantly involving T cells: Tregs (*r* = 0.46), cytotoxic cells (*r* = 0.41), and CD4^+^ T cells (*r* = 0.40), with eosinophils (*r* = 0.44), and endothelial cells (*r* = 0.40) (Fig. 4b). Storage, efflux, and catabolism showed positive correlations with most immune cell populations, though weaker than for uptake (Fig. 4b). Regarding biosynthesis, while most correlations with immune cell populations were weak, slightly stronger negative associations were seen with B cells (*r* = -0.35), neutrophils (*r* = -0.35), and plasma cells (*r* = -0.39) (Fig. 4b). Across tumor types, immune cell correlation patterns were largely consistent, except in OV tumors, where correlations with cholesterol biosynthesis were slightly positive, ranging from −0.10 for Fibroblasts to 0.32 for cytotoxic cells (Supplementary Fig. S10).

Lastly, to assess whether cholesterol-related pathways might interact or exert coordinated effects on patient survival, we investigated whether pathways associated with a favorable prognosis could modulate the negative impact of cholesterol uptake on patient prognosis. Pan-cancer samples were initially categorized into uptake-low and uptake-high groups. Each group was further stratified according to the scores of the remaining pathways, yielding corresponding high and low subgroups. Subsequently, survival analyses were performed on these subgroups. As a result, we observed that in the cholesterol uptake-low groups, there is a drastic reduction on patient survival when the catabolism is also low (p-value = 6e-07) (Supplementary Fig. S11b), while the combinations of cholesterol uptake with the other pathways have no effect on patient survival (Supplementary Fig. S11a, c, and d). To investigate the factors underlying this drastic decrease in patient survival, we conducted GSEA on uptake-low samples, comparing groups with low versus high catabolism scores. Enrichment map analysis of the catabolism-low group showed enrichment of pathways mainly related with cell proliferation and DNA replication (Supplementary Fig. S12a). Catabolism-low tumors also showed an increase of fibroblasts and endothelial cells, with immune cells following different trends. Specifically, there was a decrease in neutrophils, plasma cells, and M1-like macrophages, and an increase in cytotoxic cells and regulatory T cells (Supplementary Fig. S12b), suggestive of an immunosuppressive signature. Yet, the increase of cytotoxic T cells led us to further explore the expression of immune checkpoint genes. While most checkpoints (e.g., PDCD1, TIGIT, LAG3, CTLA4) were downregulated in the catabolism-low samples, the gene coding for the immune inhibitory receptor SIGLEC7 was upregulated (Supplementary Fig. S12c).

### Cholesterol uptake associates with an inflamed microenvironment and with poor prognosis

**Fig. 5:**
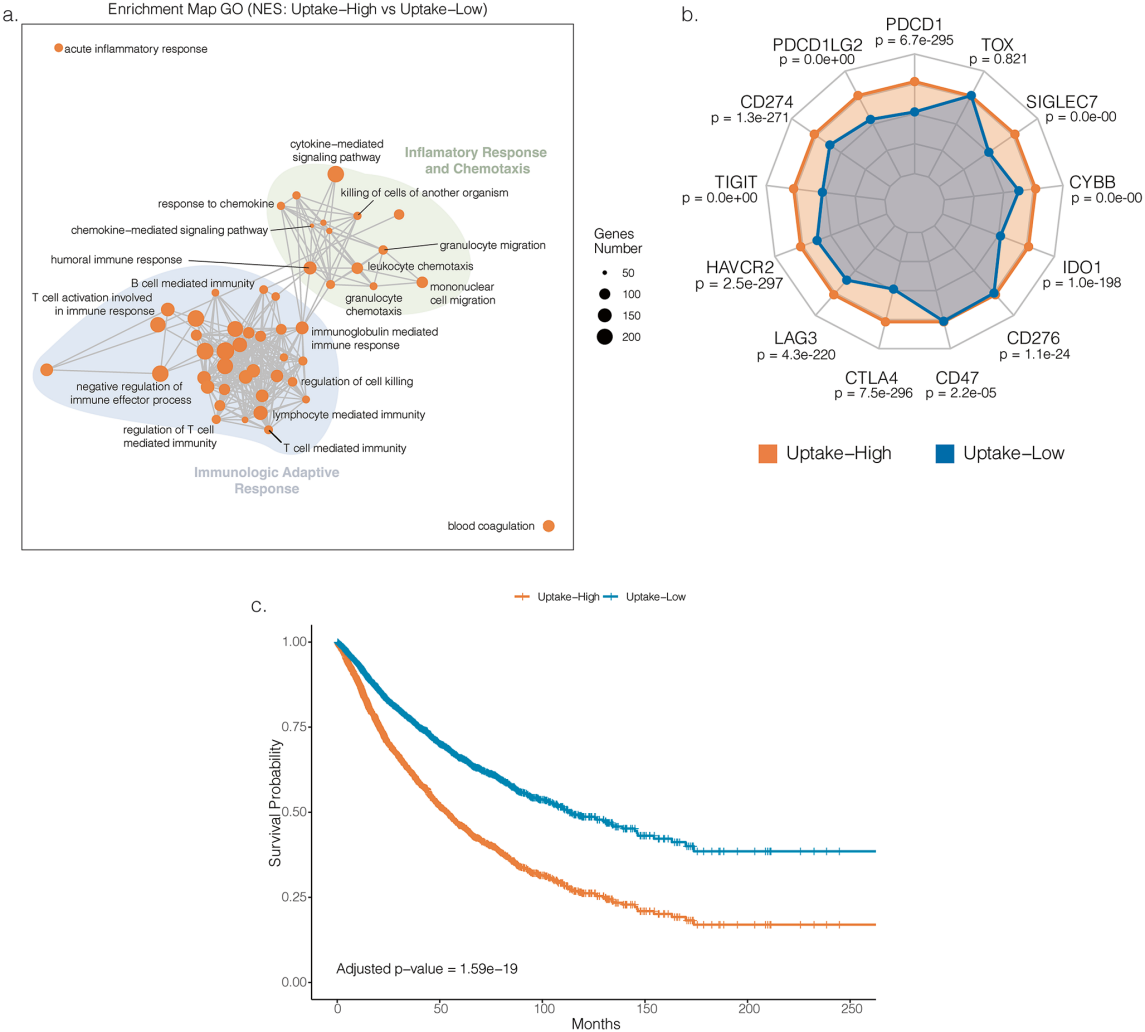
Immune contexture variation and survival outcomes between groups with distinct cholesterol uptake. **a.** Enrichment map of gene set enrichment analysis (GSEA) of uptake-low versus uptake-high samples. Node size is related to the number of components identified within a gene set. GSEA terms enriched in the uptake-high group are coloured in orange and grouped into nodes with associated terms; **b.** Expression of the immune supressor genes in both uptake-high and uptake-low groups; *p* denotes the two-sided *p*-value obtained from either a Student’s *t*-test or a Mann–Whitney *U* test, depending on data distribution, which was assessed using the Shapiro–Wilk test; **c.** Pan-cancer overall survival curves for uptake-low and high groups. Model was adjusted for the remaining cholesterol pathways and p-value was calculated using Wald test

Given consistent correlations of uptake with both tumor and microenvironment features, this pathway was further explored. For that, samples were grouped by the expression levels of uptake-related genes, and GSEA was performed to identify pathways altered in high versus low contexts. Top-50 enrichment map nodes corresponding to upregulated transcripts in the uptake-high group were exclusively associated with inflammation and immune response (Fig. 5a). While immune-related pathway enrichment persisted across uptake groups, other pathways emerged, particularly synapse-like signaling (uptake-medium versus uptake-low) and organic acid, lipid and xenobiotic metabolism (uptake-high versus uptake-medium) (Supplementary Fig. S13a and S13b, respectively).

Given that a high degree of tumor immune infiltration is commonly associated with a high tumor mutational burden (TMB), we evaluated whether tumors with high uptake also exhibited a high TMB. No significant association between cholesterol uptake and TMB was found (Supplementary Fig. S13c), thus pinpointing cholesterol uptake as an independent regulator of tumor immune infiltration.

Following the pronounced inflammation and immune infiltration in the uptake-high group, we analyzed immune-checkpoint gene expression to explore potential immune modulation pathways. A significant upregulation was observed in the uptake-high group for all the immune-checkpoints analyzed, with exception for Thymocyte selection-associated HMG-box (TOX) (Fig. 5b). Finally, given the concurrent increase in inflammation and immune-checkpoint expression, potentially reflecting activation or exhaustion, we compared overall survival between patients with high and low uptake. This analysis showed reduced survival in the high-uptake group compared with the low-uptake group (Fig. 5c).

### Cholesterol uptake positively correlates with KRAS signaling

Given the strong correlation between cholesterol uptake and KRAS signaling, along with the relevance of KRAS in cancer [26, 27], we further investigated this association. First, to assess whether cholesterol uptake associates with other cancer driver genes, we compared mutation frequencies for the ten most commonly mutated cancer genes [28] between the uptake-high and uptake-low groups. *KRAS* showed the greatest difference in mutation frequency between groups (Supplementary Fig. S14a), revealing a higher mutation rate in tumors with high cholesterol uptake: 55% of uptake-high cases versus less than 23% of uptake-low cases.

**Fig. 6:**
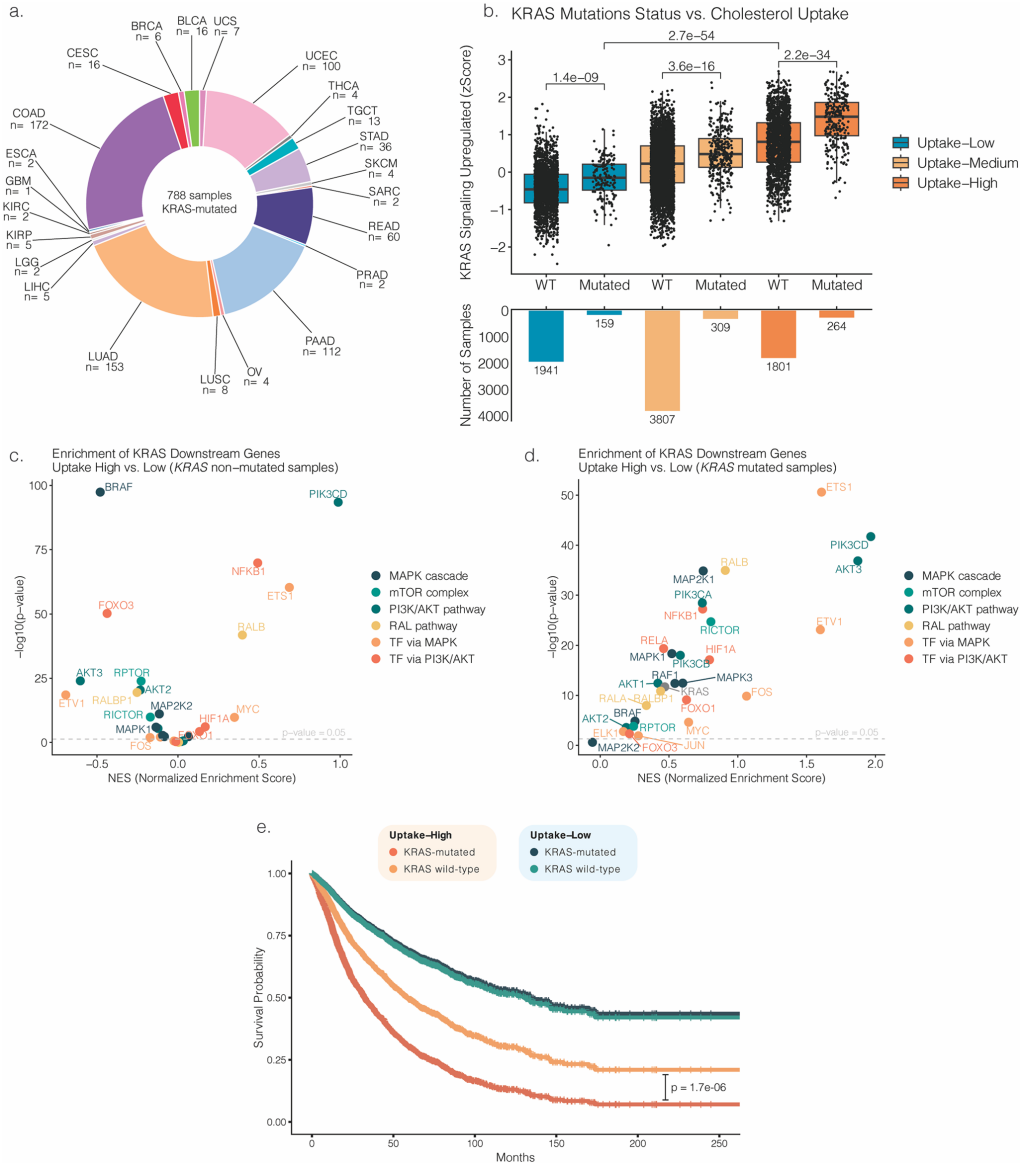
Functional association between KRAS signaling and cholesterol uptake. **a**. Frequency of the *KRAS* mutation across the TCGA projects studied; **b**. Comparison of the mean *KRAS signaling upregulated* pathway score between different uptake groups, stratified by *KRAS* mutational status; **c**. Enrichment of KRAS downstream effectors in uptake-high versus uptake-low samples, in *KRAS* wild-type samples; **d. **Same analysis performed in *KRAS*-mutated samples. *p* denotes the two-sided *p*-value obtained from either a Student’s *t*-test or a Mann–Whitney *U* test, depending on data distribution, which was assessed using the Shapiro–Wilk test; **e**. Survival analysis of the coordinated effect of cholesterol uptake and *KRAS* mutational status. Curves show overall survival differences among the resulting patient subgroups. P-values were calculated using the Wald test. Models were adjusted for the remaining cholesterol pathways

To further explore the functional association between cholesterol uptake and KRAS signaling, we compared the *KRAS signaling upregulated* score between *KRAS*-mutated and wild-type samples (Fig. 6a) according to the cholesterol uptake group (Fig. 6b). The distribution of *KRAS* mutations across tumor types included in the cohort is shown in Supplementary Figure S14b. By comparing mutated versus wild-type samples within the same uptake group, mutated samples consistently exhibited higher *KRAS signaling upregulated* scores. Notably, the comparison of uptake-low *KRAS*-mutated samples with uptake-high *KRAS* wild-type revealed significantly higher *KRAS signaling upregulated* score in the non-mutated uptake-high group. These data suggest that high cholesterol uptake drives greater expression of KRAS pathway-related genes than *KRAS* mutations do when cholesterol uptake is low. This is further supported by a strong positive correlation between cholesterol uptake and the *KRAS signaling upregulated* score in both *KRAS*-mutated and wild-type samples (Supplementary Fig. S15a and S15b), indicating that cholesterol uptake influences KRAS pathway activity independently of *KRAS* mutational status. This correlation was observed across tumor types except in ACC and LIHC, where it was not significant (Supplementary Fig. S15c).

To further investigate this association, we selected the KRAS downstream effectors from the differentially expressed genes identified in the uptake-low versus uptake-high comparison. In the *KRAS* wild-type samples, the uptake-high group showed enrichment of *PIK3CD* and *NFKB1* (a PI3K/AKT transcription factor), whereas the uptake-low samples showed enrichment of *BRAF* (Fig. 6c). Curiously, the uptake-low group had a higher mutation rate of *BRAF* than the uptake-high group (Supplementary Fig. S14a). In contrast, *KRAS*-mutated samples showed global upregulation of downstream effectors in uptake-high samples, indicating broad activation rather than a pathway-specific effect (Fig. 6d). These findings show that, unlike *KRAS*-mutated tumors, in wild-type tumors cholesterol uptake levels dictate preferentially downstream pathway engagement. To evaluate the survival impact of the interaction between uptake and *KRAS* mutation, pan-cancer multivariable Cox proportional hazards models were fitted. This analysis revealed that in the uptake-high subset, *KRAS* mutations were associated with significantly reduced patient survival. Conversely, in the uptake-low subset, *KRAS* mutations did not impact prognosis (Fig. 6e).

## Discussion

Unlike most previous studies focusing on individual cholesterol metabolism pathways within a single tumor type, our pan-cancer study provides an integrated analysis of cholesterol metabolism across cancer types. Our findings demonstrate that virtually all tumors display alterations in at least one cholesterol-related pathway, supporting cholesterol dysregulation as a hallmark of tumor biology. From this thorough analysis, cholesterol uptake emerges as a crucial contributor to tumor biology, strongly affecting pan-cancer survival.

Our results challenge the view that tumors exhibit concurrent increases in cholesterol biosynthesis, uptake, and storage, as no tumor type showed consistent and concomitant upregulation of all three pathways. In fact, some tumors, such as lung and stomach, even displayed a global decrease in most cholesterol-related pathways. Interestingly, the pathway most studied and traditionally associated with cholesterol dysregulation, biosynthesis, does not display a consistent behavior across tumor types. As shown, tumors with significant differences in cholesterol biosynthesis, half exhibited an increase versus normal tissues, while the other half showed a decrease. This variability challenges the prevailing conception of a universal upregulation of cholesterol biosynthesis [1, 29] and highlights the organ-specific nature of cholesterol metabolism remodeling. In fact, some cancer types, such as glioblastoma and clear cell renal carcinoma, exhibit cholesterol auxotrophy, as they have a lower capacity to synthesize cholesterol, depending on constitutive activation of lipoprotein uptake to survive [30, 31]. Our study not only validated these observations as it also demonstrated that tumors such as skin, thyroid, and testis, also presented the same auxotrophic behavior. In contrast, liver, breast, and intestine revealed an opposite pattern, with lower expression of cholesterol uptake genes and increased cholesterol biosynthesis gene expression. Interestingly, pancreas and uterus tumors upregulated both pathways. Such heterogeneity in the regulation of cholesterol biosynthesis among different tumor types may critically limit the use of statins as a generalized anticancer therapeutic strategy. In fact, our data are consistent with the literature, showing that simvastatin efficacy was more pronounced in bladder, breast, colon, and lung [20]. Apart from the lung, in our data, biosynthesis was the only cholesterol-related pathway that was upregulated in these organs. In lung tumors, where all cholesterol-related pathways were downregulated, any metabolic perturbation may have a stronger effect, as it cannot be compensated for alternative routes, potentially explaining the higher sensitivity. As tumor stage and histologic/molecular subtypes introduce additional layers of heterogeneity in cholesterol metabolism, this highlights the need for tumor-type–specific studies that explicitly address compensatory mechanisms across cholesterol-related pathways.

While cholesterol dysregulation appears to be a widespread feature of tumors, the extent and direction of this remodeling vary substantially among the different cholesterol-related pathways. Notably, cholesterol biosynthesis, despite extensive investigation in clinical trials, showed the highest variability across tumor types and lacked consistent association with pan-cancer patient outcome, revealing limited prognostic significance and a weak association with tumor features. In the literature, numerous studies describe cholesterol biosynthesis as having a critical role in tumor progression and immune modulation [32, 33]. In our finding this effect was not observed, however this apparent discrepancy with previous studies may stem from the use of bulk transcriptomic data, where signals from multiple cell types within the tumor microenvironment can dilute tumor cell–intrinsic biosynthesis signatures. Nevertheless, tumor-bulk analysis accounts for the contribution of different tumor cellular components, which are known to play an important role in tumor progression and in this study, in particular, allowed to identify an association between cholesterol uptake and the immune counterpart. Furthermore, cholesterol uptake displayed homogenous scores across tumors, reaching variability levels comparable to those observed in normal tissues. Remarkably, upregulation of cholesterol uptake was strongly associated with patient poorer survival. Importantly, cholesterol uptake showed the strongest correlations with cell signaling and immune regulation. Together, these associations highlight a key role of cholesterol uptake in tumor biology, promoting aggressiveness via inflammation and KRAS signaling. Thus, these associations suggest that cholesterol uptake is not merely a metabolic adaptation but a driver of the tumor phenotype itself.

Inflammatory signaling is a critical component of tumor development, influencing proliferation, immune evasion, and response to therapy [34]. The association between inflammatory signaling and cholesterol dysregulation is well recognized and increasingly appreciated in cancer [11, 12]. In our study, high cholesterol uptake was consistently associated with upregulation of key inflammatory pathways, including IL6/JAK/STAT3, complement system, and interferon-gamma responses, suggesting that cholesterol uptake may orchestrate a pro-inflammatory tumor microenvironment. These findings align with previous studies showing that cholesterol accumulation in immune cells drives pro-inflammatory responses, establishing a self-sustaining cycle of inflammation [35]. Additionally, supporting the observation that uptake-high tumors display higher immune-checkpoint molecules expression, cholesterol accumulation in immune cells also promotes an immunosuppressive phenotype that spans both innate and adaptive immune compartments [14, 35]. However, both cancer cells and immune cells can take up cholesterol. Thus, given that our results derive from bulk RNA-Seq data, we cannot determine whether tumor cells or immune cells are the main contributors to the higher cholesterol uptake gene expression. These findings suggest that cholesterol uptake may actively shape the immune landscape, fostering a pro-tumorigenic microenvironment, while targeting it may enhance responsiveness to immunotherapy. Further studies are needed to determine whether targeting cholesterol uptake, alone or in combination with immune-checkpoint blockade can reprogram tumor-associated inflammation to improve patient survival.

Our data revealed a positive association between cholesterol uptake and *KRAS* mutations/signaling, which impacts patient prognosis, although the direction of regulation remains to be determined. The interplay between KRAS and cholesterol metabolism in cancer has been described in multiple contexts, highlighting a bidirectional relationship [26, 27, 36–38]. For instance, *KRAS* mutations induce SREBP transcription, promoting cholesterol synthesis and uptake in breast cancer, and upregulate GRAMD1A, which mediates cholesterol transport to the endoplasmic reticulum, in colorectal cancer [37, 38]. Similarly, in *KRAS*-mutant pancreatic cancer, LDL receptor expression increases across disease stages, correlating with higher recurrence rates [36]. Additionally, in *KRAS*-mutant lung cancer, disruption of cholesterol efflux in epithelial progenitor cells increased intracellular cholesterol, expanded tumor progenitors, and promoted an immunosuppressive tumor microenvironment [39]. It is well established that *KRAS* mutations are associated with increased levels of macropinocytosis, one of the routes that facilitates cholesterol uptake [40]. Additionally, in pancreatic cancer, mutant *KRAS* is associated with increased of ADP-ribosylation factor 6 (ARF6), a key regulator of clathrin-mediated endocytosis and late endosomes trafficking [41]. Interestingly, LDL clathrin-mediated endocytosis represents another major pathway for cholesterol uptake [42]. Together, these mechanisms provide a plausible explanation for the correlation between elevated KRAS signaling and enhanced uptake, as *KRAS*-mutant cells upregulate endocytic processes that promote cholesterol internalization. Favoring a mutant *KRAS*-enhanced cholesterol uptake hypothesis, we also observed higher prevalence of *KRAS*-mutant cases in uptake-high samples. However, we observed that uptake-high samples lacking *KRAS* mutations express even higher KRAS signaling-associated genes than uptake-low samples harboring *KRAS* mutations. Hence, these observations strongly suggest that cholesterol uptake also likely plays a role in regulating KRAS signaling. Particularly, the subcellular localization of KRAS, at the plasma membrane or endosomes, shapes both the type and duration of downstream signaling. Membrane-localized KRAS preferentially signals via RAF-MEK-ERK, whereas endosomal KRAS extends signal duration and favors PI3K and Ral pathways [40]. Our results, combined with the predominant endosomal trafficking of cholesterol uptake [43], suggest that the high uptake may relocate KRAS from the membrane to late endosomes, thereby enhancing KRAS-mediated PI3K signaling. Moreover, the proximity of KRAS to its activators in endosomes, can sensitize the activation, along with a sustained signaling in late endosomes [40], helps explain the strong association between cholesterol uptake and elevated KRAS signaling, without a similar association with EGF-EGFR. However, other reports show that increased cholesterol uptake elevates plasma membrane cholesterol, enhancing flexibility of membrane-bound KRAS. This flexibility improves accessibility of critical switch loops to downstream effectors, both in *KRAS*-mutant and wild-type, sustaining KRAS signaling [44]. Thus, while KRAS subcellular localization dictates distinct signaling dynamics, cholesterol uptake appears to amplify KRAS signaling in both compartments, i.e., enhancing membrane-associated signaling and sustaining endosomal signaling for prolonged activation. Therefore, it is possible that mutant *KRAS* tumors have a high dependency on specific cholesterol metabolic pathways. On the one hand, they may depend on basal levels of mevalonate pathway which produces isoprenoids such as farnesyl pyrophosphate and geranylgeranyl pyrophosphate (FPP/GGPP) needed for membrane localization of KRAS. On the other hand, high uptake amplifies KRAS signaling and sustains the high anabolic demands imposed by KRAS-driven proliferative programs, including increased membrane biogenesis and remodeling. Notably, perhexiline maleate, which suppresses the transcript levels of *Srebf2* and inhibits cholesterol uptake, decreased cancer growth and induced cell death on pancreatic organoids carrying the *Kras*G12D mutation, both in vitro and in vivo [21]. Likewise, blocking of LDLR reduced proliferation and ERK1/2 activation [45]. Conversely, PCSK9, a key inhibitor of cholesterol uptake, promoted the growth of *APC/KRAS*-mutant colorectal cancer, and its inhibition significantly reduced tumor growth both in vitro and in vivo [46]. Nonetheless, the adverse prognostic impact of high cholesterol uptake may be mediated, at least in part, by immune suppression, which could be particularly detrimental when *KRAS*-mutant cancer cells coexist with a cholesterol-rich, high-uptake microenvironment. Altogether, these findings suggest that targeting cholesterol uptake could represent a promising therapeutic strategy for both *KRAS*-mutant and for *KRAS* wild-type. Nonetheless, our data, suggest that targeting cholesterol uptake alongside KRAS inhibition in *KRAS*-mutant tumors could improve patient survival and prevent resistance from signaling rewiring and pathway reactivation.

This integrative transcriptomic analysis across large patient cohorts identified robust molecular patterns associated with distinct cholesterol-related pathways. However, because this study relies exclusively on bioinformatic analyses, functional validation of these findings is required. Moreover, single-cell approaches will be essential to overcome the inherent limitations of bulk RNA sequencing in resolving cell-type–specific contributions to the observed effects. Nonetheless, this work provides testable hypotheses regarding the role of cholesterol metabolism in tumourigenesis across different cancer types.

## Conclusion

Our systematic analysis reveals that cholesterol metabolism is extensively remodeled in a tissue- and pathway-specific manner, with cholesterol uptake emerging as a determinant of tumor aggressiveness and immune microenvironment composition. These findings delineate potential links between cholesterol metabolism, KRAS signaling, and inflammatory pathways, and highlight cholesterol uptake as a promising therapeutic vulnerability with potential to enhance responses to immunotherapy and KRAS-targeted treatments. Further studies that account for tissue-specific metabolic context are essential to refine therapeutic strategies aimed at modulating cholesterol uptake in cancer.

## Supplementary Information

Below is the link to the electronic supplementary material.

Supplementary Material 1

## Data Availability

The data analyzed in this study were obtained from The Cancer Genome Atlas (TCGA) [https://portal.gdc.cancer.gov/] using the TCGAbiolinks R package (v2.31.3), and from GTEx Portal [https://gtexportal.org/home/downloads/adult-gtex/bulk_tissue_expression], specifically from brain (Cerebellar Hemisphere, Cortex, and Frontal Cortex (BA9)), pancreas, ovarian, testis, and skin.
