# Supplementary material for: Pan-cancer analysis of cholesterol metabolism reveals the uptake as a modulator of tumor immune features and of the KRAS pathway
Source: Cell Oncol (Dordr). 2026 Feb 12;49(1):45. doi: 10.1007/s13402-026-01171-z (PMC12901284; doi:10.1007/s13402-026-01171-z)
Supplement: Supplementary file 1 — Supplementary Material 1 [file 13402_2026_1171_MOESM1_ESM.pdf]

Table S1. Cholesterol-associated Gene Ontology (GO) pathways selected for manual curation and used as references in the definition of cholesterol metabolism gene sets.

Pathway	Reference GO pathways
Biosynthesis	GO_POSITIVE_REGULATION_OF_CHOLESTEROL_BIOSYNTHETIC_PROCESS
Catabolism	GO_CHOLESTEROL_CATABOLIC_PROCESS
Efflux	GO_POSITIVE_REGULATION_OF_CHOLESTEROL_EFFLUX
Storage	GO_POSITIVE_REGULATION_OF_CHOLESTEROL_STORAGE
Uptake	GO_RECEPTOR_MEDIATED_ENDOCYTOSIS_INVOLVED_IN_CHOLESTEROL_TRANSPORT

Table S2. Gene sets used to assess tumor-related traits across cancer types.

HALLMARK_ANGIOGENESIS	HALLMARK_P53_PATHWAY
HALLMARK_APOPTOSIS	HALLMARK_PEROXISOME
HALLMARK_COMPLEMENT	HALLMARK_PI3K_AKT_MTOR_SIGNALING
HALLMARK_DNA_REPAIR	HALLMARK_PROTEIN_SECRETION
HALLMARK_E2F_TARGETS	HALLMARK_REACTIVE_OXYGEN_SPECIES_PATHWAY
HALLMARK_EPITHELIAL_MESENCHYMAL_TRANSITION	HALLMARK_TGF_BETA_SIGNALING
HALLMARK_FATTY_ACID_METABOLISM	HALLMARK_TNFA_SIGNALING_VIA_NFKB
HALLMARK_G2M_CHECKPOINT	HALLMARK_UNFOLDED_PROTEIN_RESPONSE
HALLMARK_GLYCOLYSIS	HALLMARK_WNT_BETA_CATENIN_SIGNALING
HALLMARK_HEDGEHOG_SIGNALING	GOBP_MACROPINOCYTOSIS
HALLMARK_HEME_METABOLISM	GOBP_MEMBRANE_BIOGENESIS
HALLMARK_HYPOXIA	GOBP_MEMBRANE_DISASSEMBLY
HALLMARK_IL2_STAT5_SIGNALING	GOBP_MEMBRANE_DOCKING
HALLMARK_IL6_JAK_STAT3_SIGNALING	GOBP_MEMBRANE_RAFT_ASSEMBLY
HALLMARK_INFLAMMATORY_RESPONSE	REACTOME_ENERGY_DEPENDENT_REGULATION_OF_MTOR_BY_LKB1_AMPK
HALLMARK_INTERFERON_ALPHA_RESPONSE	REACTOME_SIGNALLING_TO_ERKS
HALLMARK_INTERFERON_GAMMA_RESPONSE	SA_PTEN_PATHWAY
HALLMARK_KRAS_SIGNALING_DN	GOBP_VESICLE_BUDDING_FROM_MEMBRANE
HALLMARK_KRAS_SIGNALING_UP	WP_EGFEGFR_SIGNALING_PATHWAY
HALLMARK_MITOTIC_SPINDLE	WP_ERBB_SIGNALING_PATHWAY
HALLMARK_MTORC1_SIGNALING	WP_P38_MAPK_SIGNALING_PATHWAY
HALLMARK_MYC_TARGETS_V1	WP_VEGFAVEGFR2_SIGNALING
HALLMARK_MYC_TARGETS_V2	GOBP_REGULATION_OF_AUTOPHAGY
HALLMARK_NOTCH_SIGNALING	GOBP_EXTRACELLULAR_EXOSOME_ASSEMBLY
HALLMARK_OXIDATIVE_PHOSPHORYLATION	GOBP_FERROPTOSIS

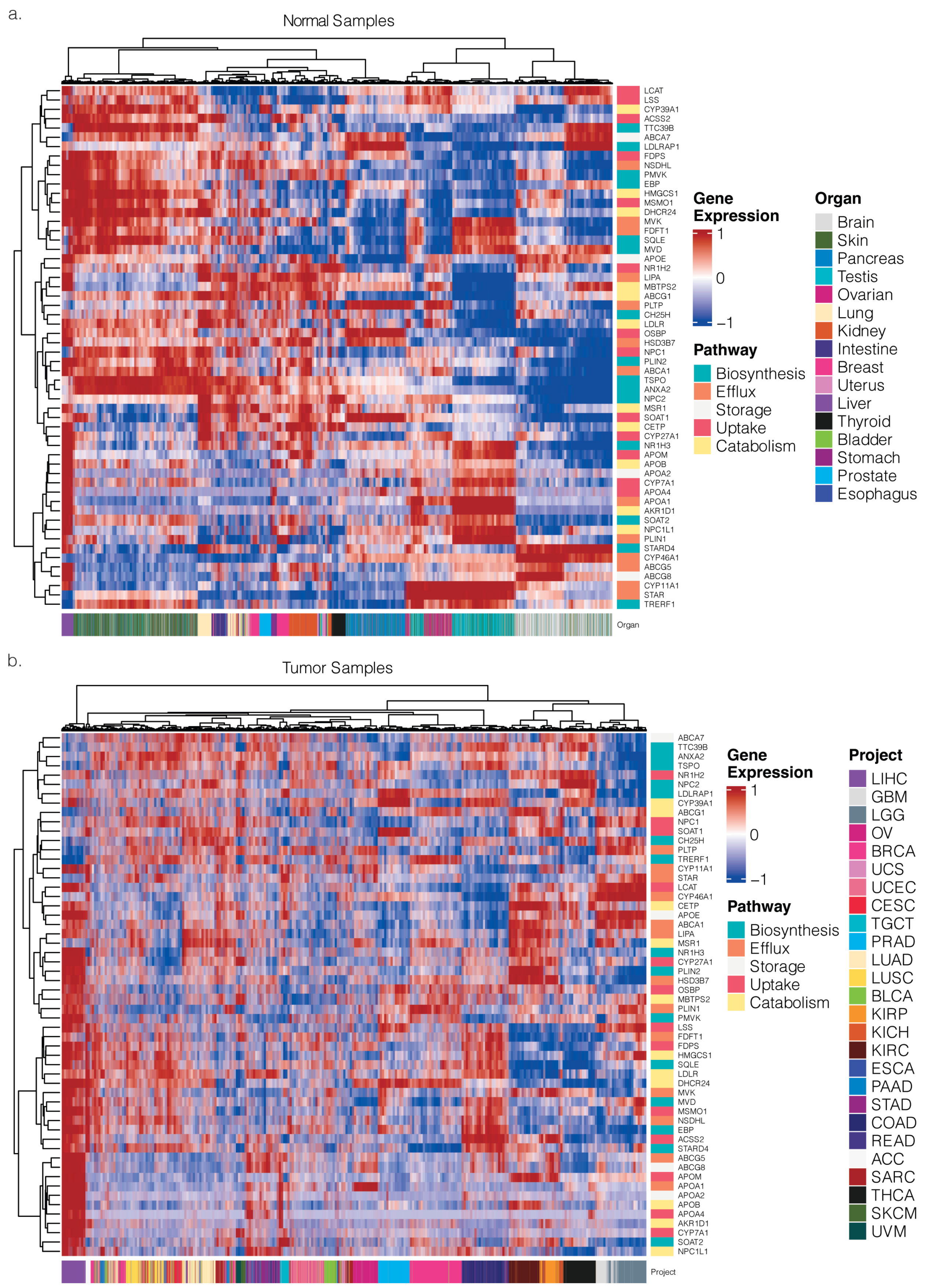

Figure S1. Gene expression heatmap, standardized by gene-wise z-scores, showing the profiles of the manually curated cholesterol metabolism pathways across normal and tumor samples for: a. Normal samples; **b.** Tumor samples across distinct organs. ACC, adrenocortical carcinoma; BLCA, bladder carcinoma; BRCA, breast carcinoma; CESC, cervical carcinoma; COAD, colon adenocarcinoma; ESCA, oesophageal carcinoma; GBM, glioblastoma multiforme; KICH, kidney chromophobe carcinoma; KIRC, kidney renal clear cell carcinoma; KIRP, kidney renal papillary carcinoma; LGG, low grade glioma; LIHC, hepatocellular carcinoma; LUAD, lung adenocarcinoma; LUSC, lung squamous cell carcinoma; PAAD, pancreatic carcinoma; PRAD, prostate carcinoma; READ, rectal adenocarcinoma; OV, ovarian serous adenocarcinoma; SARC, sarcoma; SKCM, skin melanoma; STAD, stomach carcinoma; TGCT, testicular germ cell tumor; THCA, thyroid carcinoma; UCEC, uterine carcinoma; UCS, uterine carcinosarcoma; UVM, uveal melanoma.

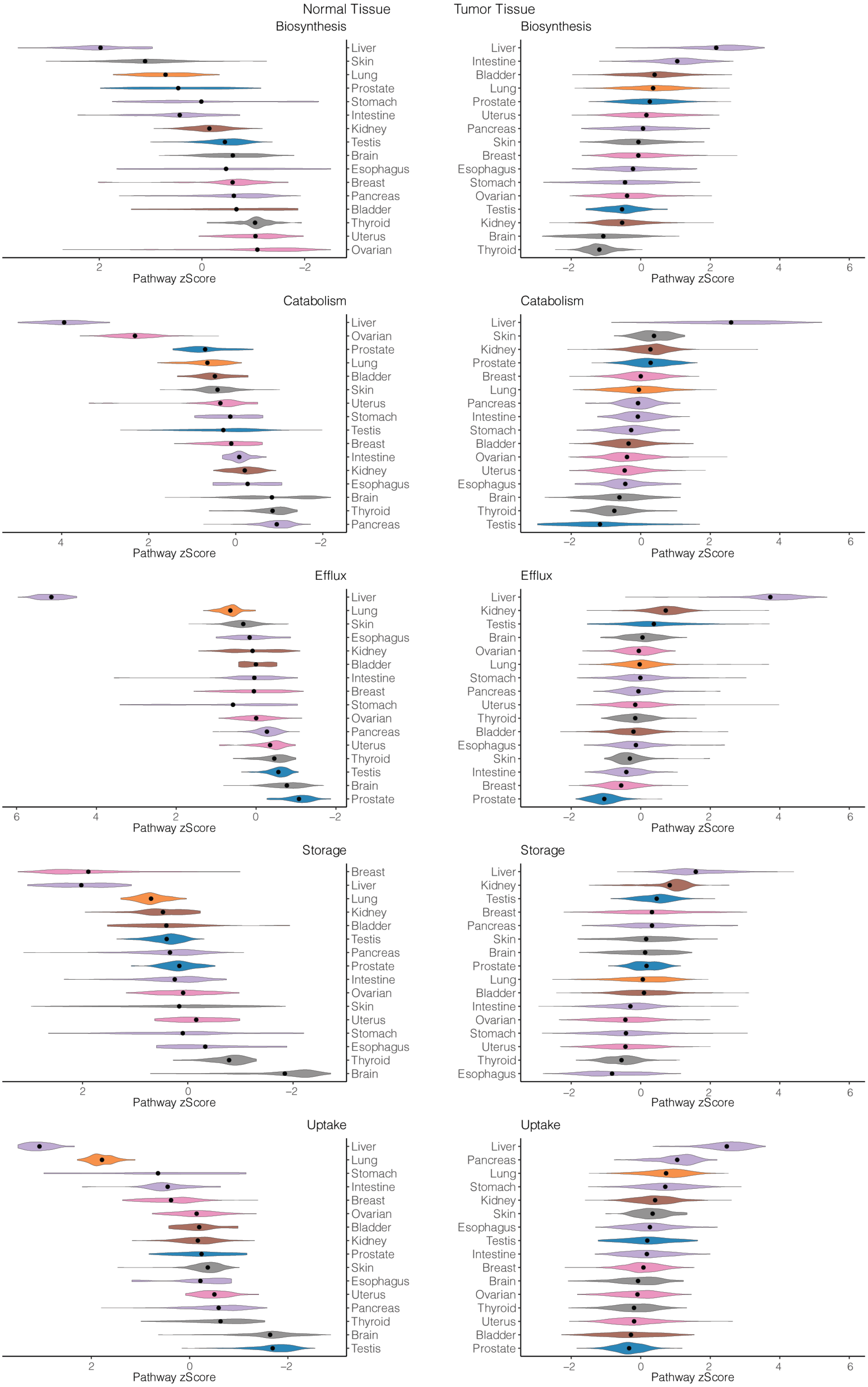

Figure S2. Comparison of cholesterol metabolism across normal and tumor tissues from multiple organs. Cholesterol pathway scores of the five cholesterol-related pathways in the normal samples, on the left, and tumor samples, on the right. The different TCGA projects were grouped considering tumor location. Organs are displayed in descending order of mean values (from top to bottom).

Table S3. Overview of tumor–normal differences in cholesterol-related pathways across tumor locations. The table reports the percentage of tumor types with significant upegulation, from those that presented significant differences. Only significant comparisons were retained.

Pathway	% Tumors upregulated	Tendency
Biosynthesis	50%	Variable
Catabolism	20%	Decreased
Efflux	46%	Variable
Storage	27%	Decreased
Uptake	67%	Increased

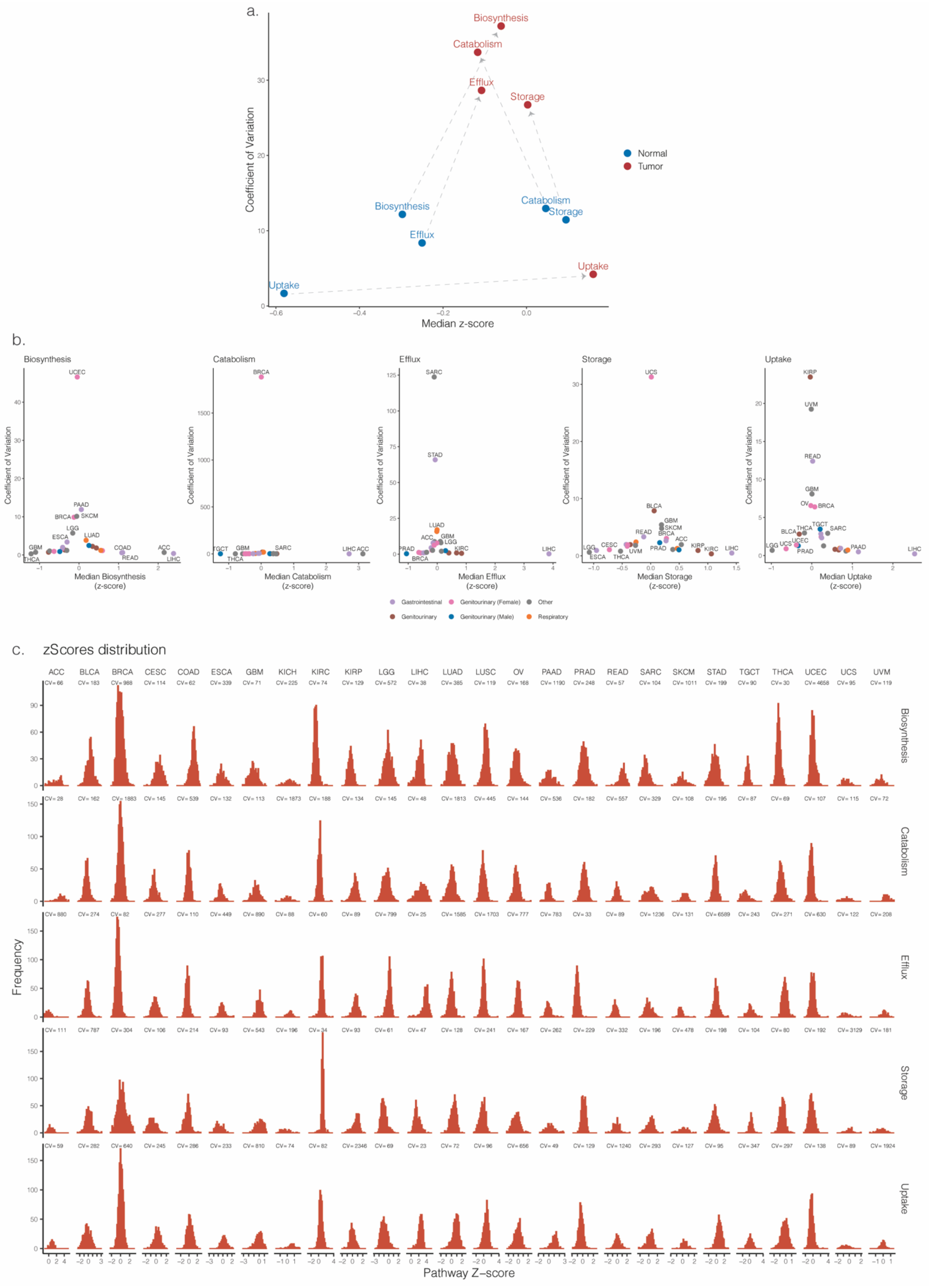

Figure S3. Pan-Cancer expression patterns of cholesterol pathways. a. Boxplot showing pan-cancer median cholesterol pathways scores and the coefficient of variation. A single dot represents a unique cholesterol-pathway value for normal tissue (in blue) and tumor tissue (in red); **b.** Boxplot showing pan-cancer median cholesterol pathways tumor scores for each cancer type. A single dot represent a unique TCGA project; **c.** Distribution of cholesterol pathways scores across TCGA tumor samples.

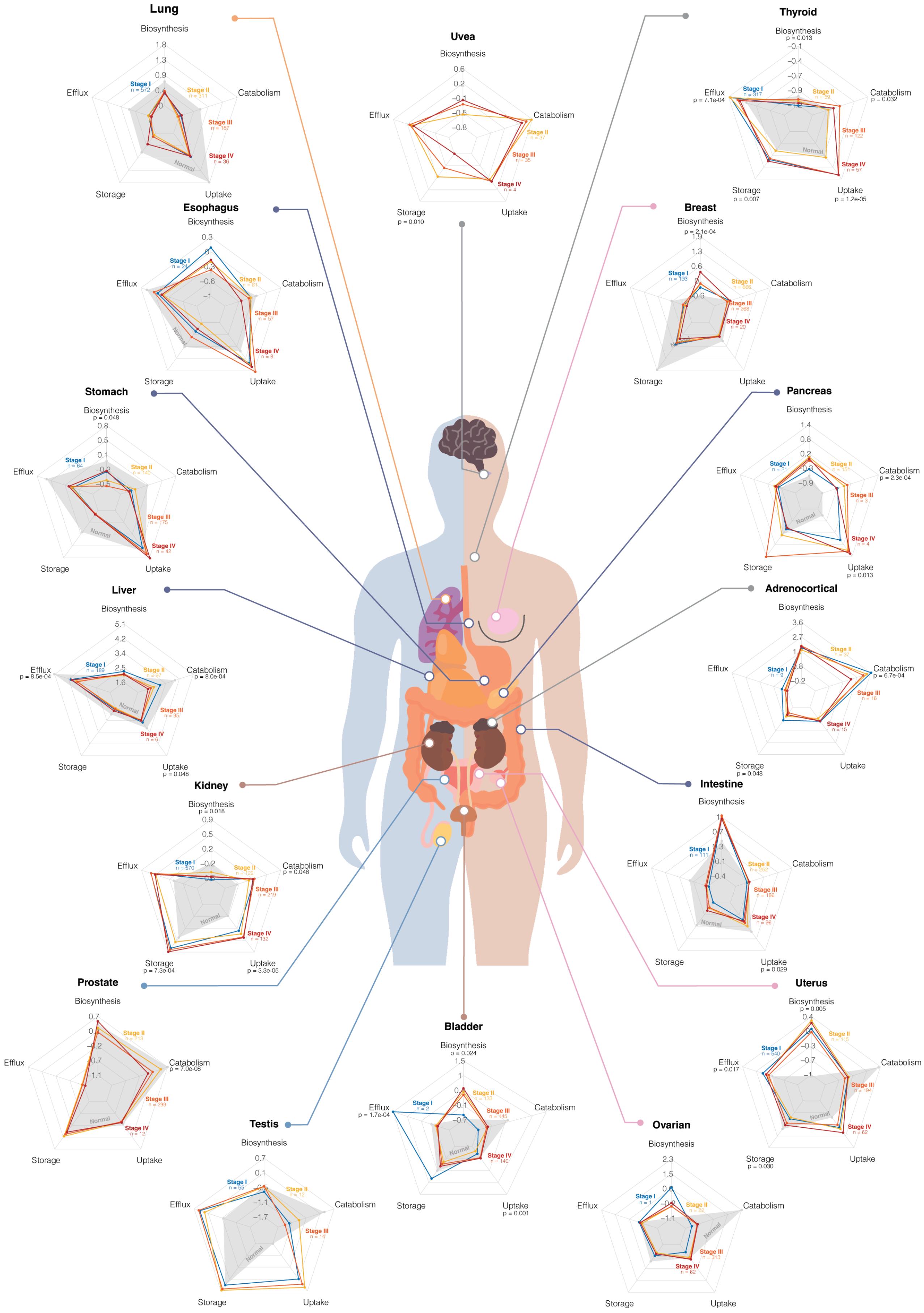

Figure S4. Comparison of scores for cholesterol-related pathways across different tumor stages within each organ of origin. The score of the corresponding normal tissue samples is shown in grey as a reference. *n* refers to the number of samples. *p* denotes the two-sided *p*-value obtained from either one-way ANOVA or Kruskal–Wallis test, depending on data distribution. Normal samples were not considered in the statistical analysis. Brain and skin samples lacked tumor stage information.

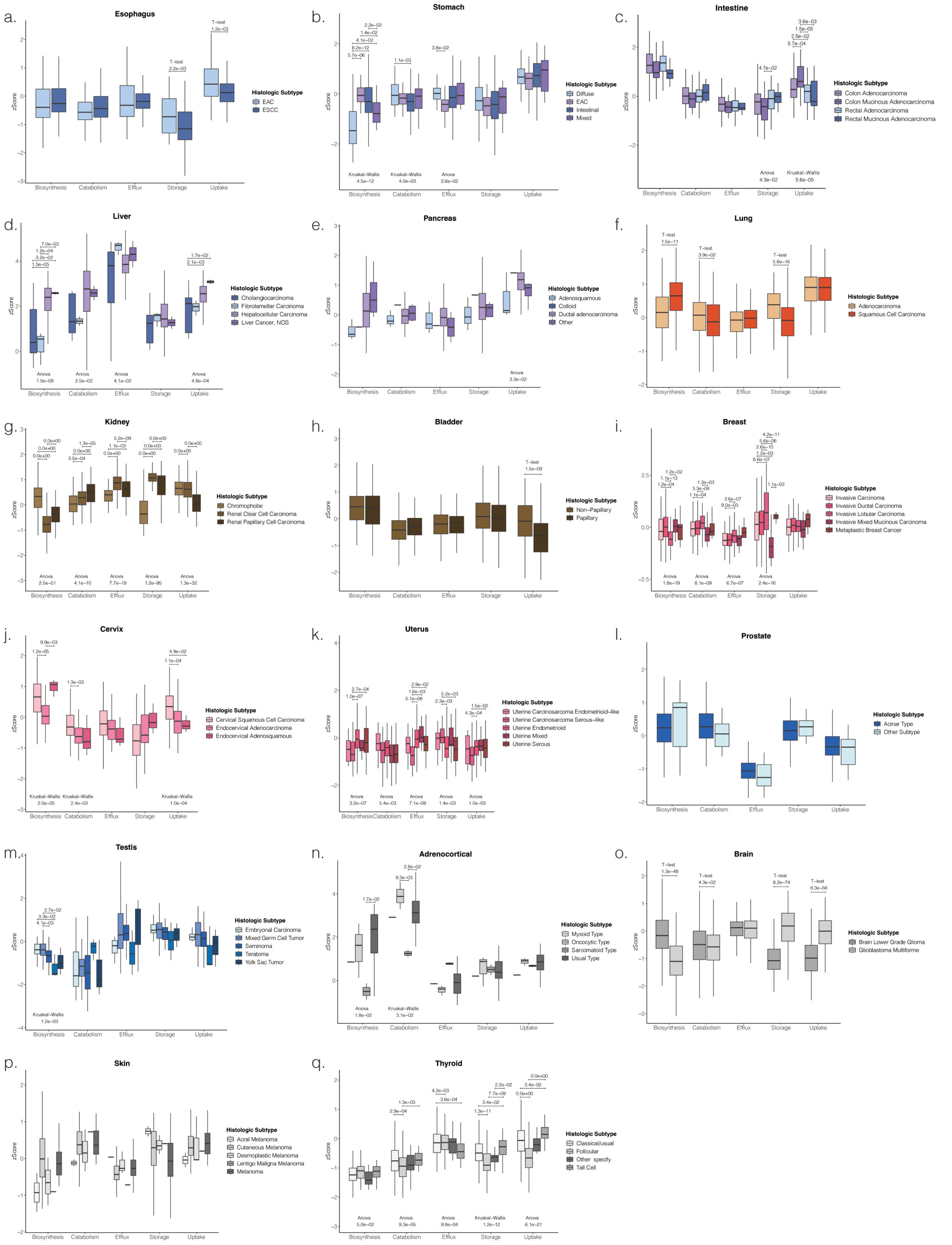

Figure S5. Comparison of ssGSEA scores for cholesterol-related pathways across the different histological subtypes of: a. Esophagus; **b.** Stomach; **c.** Intestine; **d.** Liver; **e.** Pancreas; **f.** Lung; **g.** Kidney; **h.** Bladder; **i.** Breast; **j.** Cervix; **k.** Uterus; **l.** Prostate; **m.** Testis; **n.** Adrenocortical; **o.** Brain; **p.** Skin; **q.** Thyroid. Statistical significance was assessed using ANOVA or Kruskal–Wallis tests, followed by appropriate post hoc analyses (Tukey's HSD or Dunn's test) depending on data distribution. Histological subtype data were not available for Uvea and Ovarian.

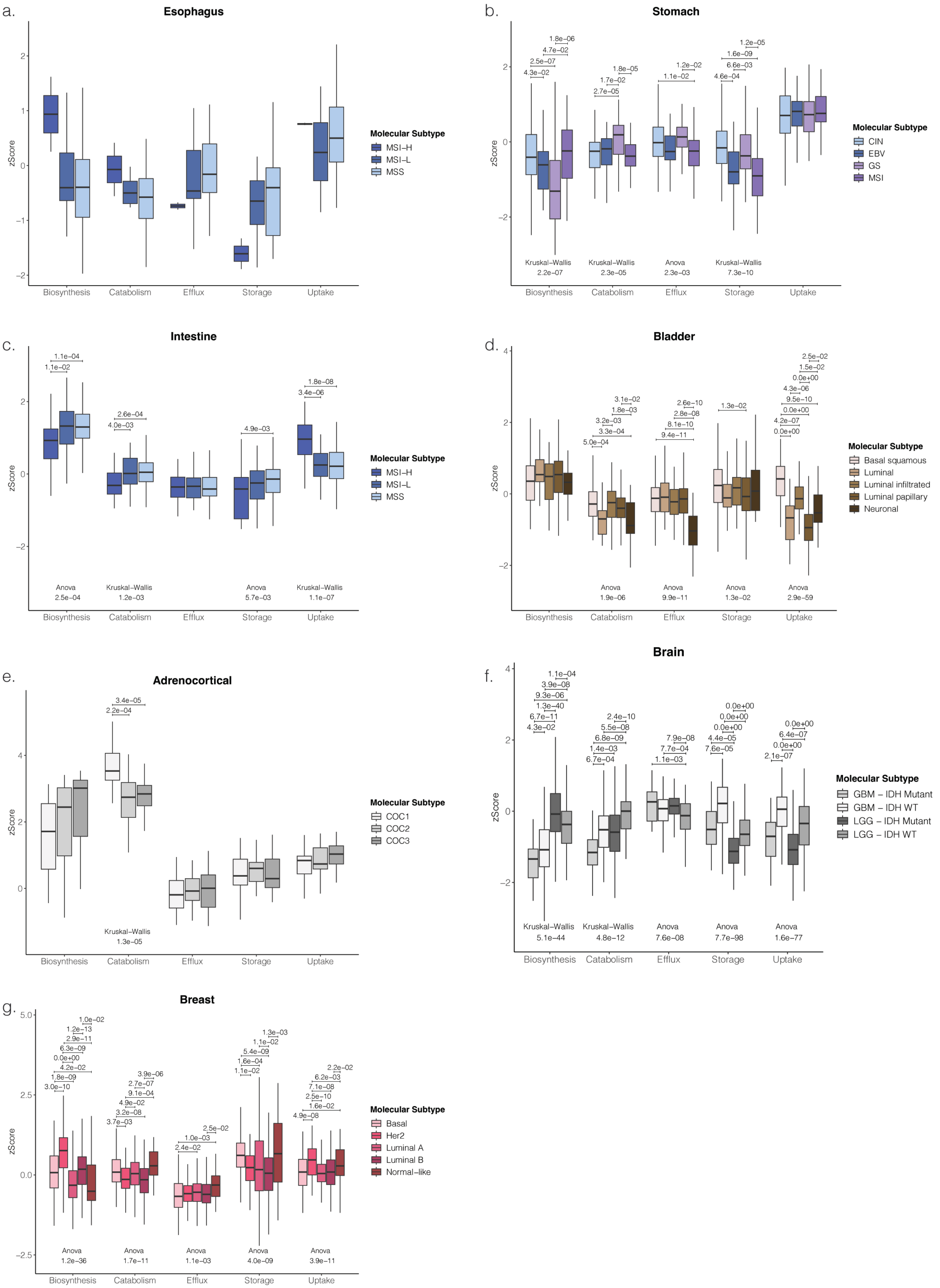

Figure S6. Comparison of gene expression of cholesterol-related pathway, measured by ssGSEA scores, across the molecular subtypes of: a. Esophagus; **b.** Stomach; **c.** Intestine; **d.** Bladder; **e.** Adrenocortical; **f.** Brain; **g.** Breast. Statistical significance was assessed using ANOVA or Kruskal–Wallis tests, followed by post hoc analysis with Tukey's HSD or Dunn's test, depending on data distribution. Molecular subtype data was unavailable for organs of origin that were not specified.

a.

Project	Samples	P–Value	Hazard Ratio
ACC	76	0.91	1.03 (0.64 – 1.65)
BLCA	399	0.96	0.99 (0.8 – 1.24)
BRCA	1048	0.60	1.08 (0.82 – 1.42)
CESC	280	0.33	1.22 (0.81 – 1.84)
COAD	421	0.19	0.81 (0.59 – 1.11)
ESCA	156	0.34	1.2 (0.82 – 1.77)
**GBM**	**256**	**0.02**	**0.78 (0.63 – 0.97)**
KICH	65	0.98	1.01 (0.34 – 3)
KIRC	504	0.20	0.82 (0.6 – 1.11)
KIRP	252	0.35	0.64 (0.25 – 1.62)
**LGG**	**444**	**0.01**	**0.66 (0.49 – 0.9)**
LIHC	340	0.50	1.12 (0.8 – 1.56)
LUAD	492	0.32	1.12 (0.89 – 1.41)
LUSC	469	0.85	0.98 (0.78 – 1.23)
OV	392	0.33	0.89 (0.71 – 1.12)
PAAD	174	0.86	1.03 (0.73 – 1.46)
PRAD	480	0.80	1.14 (0.42 – 3.13)
READ	153	0.77	1.11 (0.54 – 2.28)
SKCM	73	0.18	0.63 (0.32 – 1.23)
STAD	378	0.84	1.02 (0.84 – 1.25)
THCA	490	0.49	0.53 (0.08 – 3.27)
UCEC	521	0.55	1.13 (0.76 – 1.66)
UCS	57	0.34	0.65 (0.27 – 1.57)
UVM	76	0.53	1.43 (0.47 – 4.38)

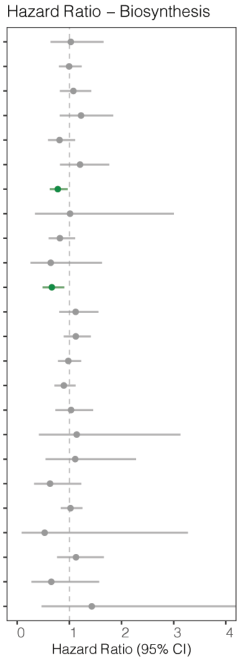

b.

Project	Samples	P–Value	Hazard Ratio
**ACC**	**76**	**0.00**	**0.39 (0.22 – 0.69)**
BLCA	399	0.88	1.02 (0.77 – 1.35)
**BRCA**	**1048**	**0.00**	**0.65 (0.48 – 0.87)**
CESC	280	0.13	0.68 (0.41 – 1.12)
COAD	421	0.81	0.95 (0.6 – 1.5)
ESCA	156	0.26	1.29 (0.83 – 2.01)
GBM	256	0.94	0.99 (0.72 – 1.35)
**KICH**	**65**	**0.00**	**0.1 (0.02 – 0.47)**
KIRC	504	0.73	1.08 (0.71 – 1.63)
**KIRP**	**252**	**0.05**	**0.48 (0.23 – 1.01)**
LGG	444	0.19	0.8 (0.57 – 1.11)
**LIHC**	**340**	**0.01**	**0.77 (0.64 – 0.93)**
LUAD	492	0.67	0.95 (0.74 – 1.21)
LUSC	469	0.86	1.02 (0.81 – 1.3)
OV	392	0.46	1.08 (0.87 – 1.34)
PAAD	174	0.70	0.89 (0.48 – 1.63)
**PRAD**	**480**	**0.02**	**0.18 (0.04 – 0.78)**
READ	153	0.27	1.87 (0.61 – 5.71)
SKCM	73	0.44	1.47 (0.56 – 3.86)
STAD	378	0.35	1.17 (0.84 – 1.62)
THCA	490	0.70	1.26 (0.39 – 4.06)
UCEC	521	1.00	1 (0.66 – 1.51)
UCS	57	0.34	1.39 (0.71 – 2.73)
**UVM**	**76**	**0.01**	**0.1 (0.02 – 0.55)**

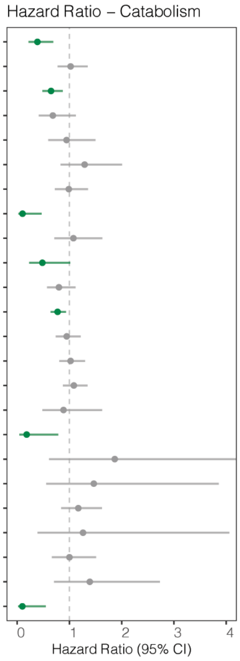

c.

Project	Samples	P–Value	Hazard Ratio
ACC	76	0.71	1.17 (0.52 – 2.61)
**BLCA**	**399**	**0.00**	**0.6 (0.44 – 0.82)**
BRCA	1048	0.60	1.13 (0.71 – 1.8)
CESC	280	0.08	0.63 (0.38 – 1.06)
COAD	421	0.39	1.24 (0.76 – 2.02)
ESCA	156	0.43	1.24 (0.72 – 2.13)
**GBM**	**256**	**0.00**	**0.58 (0.4 – 0.84)**
**KICH**	**65**	**0.00**	**0 (0 – 0.01)**
KIRC	504	0.73	0.94 (0.64 – 1.37)
**KIRP**	**252**	**0.02**	**0.33 (0.13 – 0.82)**
**LGG**	**444**	**0.01**	**0.57 (0.38 – 0.85)**
LIHC	340	0.22	0.84 (0.63 – 1.11)
LUAD	492	0.12	0.75 (0.52 – 1.08)
LUSC	469	0.20	0.79 (0.55 – 1.13)
OV	392	0.35	0.86 (0.62 – 1.18)
PAAD	174	0.20	0.73 (0.46 – 1.17)
PRAD	480	0.36	2.62 (0.33 – 20.59)
READ	153	0.43	1.67 (0.46 – 6.06)
**SKCM**	**73**	**0.04**	**0.22 (0.05 – 0.93)**
STAD	378	0.20	1.22 (0.9 – 1.66)
THCA	490	0.56	0.61 (0.12 – 3.2)
UCEC	521	0.72	0.93 (0.62 – 1.4)
UCS	57	0.30	1.38 (0.75 – 2.55)
UVM	76	0.28	0.44 (0.1 – 1.93)

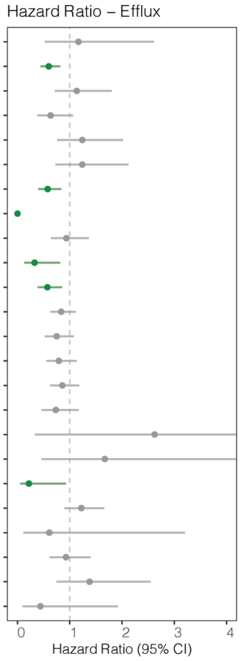

d.

Project	Samples	P–Value	Hazard Ratio
ACC	76	0.41	0.68 (0.28 – 1.69)
BLCA	399	0.54	0.93 (0.74 – 1.17)
BRCA	1048	0.46	0.93 (0.76 – 1.13)
CESC	280	0.09	1.38 (0.95 – 2.01)
COAD	421	0.96	1.01 (0.74 – 1.37)
ESCA	156	0.40	1.22 (0.77 – 1.92)
GBM	256	0.32	0.88 (0.68 – 1.14)
KICH	65	0.89	1.08 (0.36 – 3.28)
KIRC	504	0.76	0.92 (0.52 – 1.61)
KIRP	252	0.84	1.07 (0.56 – 2.02)
LGG	444	0.20	1.35 (0.86 – 2.12)
LIHC	340	0.62	0.93 (0.7 – 1.23)
**LUAD**	**492**	**0.04**	**1.38 (1.02 – 1.86)**
LUSC	469	0.11	1.21 (0.96 – 1.53)
OV	392	0.39	0.92 (0.77 – 1.11)
PAAD	174	0.31	0.84 (0.61 – 1.17)
PRAD	480	0.49	0.52 (0.08 – 3.43)
READ	153	0.64	0.85 (0.43 – 1.68)
SKCM	73	0.95	1.02 (0.55 – 1.9)
**STAD**	**378**	**0.02**	**1.29 (1.04 – 1.6)**
THCA	490	0.49	0.65 (0.19 – 2.23)
UCEC	521	0.45	1.14 (0.81 – 1.59)
UCS	57	0.66	1.2 (0.53 – 2.74)
UVM	76	0.65	0.79 (0.29 – 2.18)

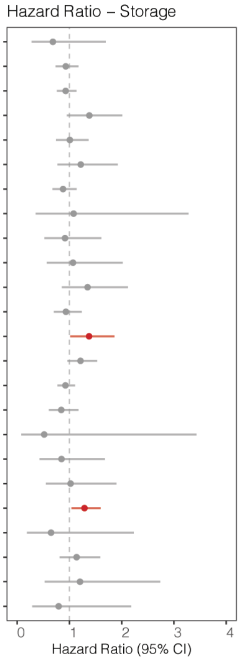

e.

Project	Samples	P–Value	Hazard Ratio
ACC	76	0.86	1.13 (0.29 – 4.36)
**BLCA**	**399**	**0.00**	**1.39 (1.11 – 1.74)**
BRCA	1048	0.74	0.93 (0.6 – 1.43)
CESC	280	0.71	1.1 (0.67 – 1.81)
COAD	421	0.19	1.26 (0.89 – 1.78)
ESCA	156	0.87	0.96 (0.55 – 1.67)
**GBM**	**256**	**0.01**	**1.66 (1.11 – 2.48)**
**KICH**	**65**	**0.00**	**21.7 (5.32 – 88.55)**
KIRC	504	0.08	1.39 (0.97 – 2)
**KIRP**	**252**	**0.00**	**8.17 (3.12 – 21.39)**
**LGG**	**444**	**0.00**	**2.13 (1.34 – 3.38)**
LIHC	340	0.12	1.48 (0.91 – 2.43)
LUAD	492	0.72	0.94 (0.69 – 1.3)
LUSC	469	0.21	1.25 (0.88 – 1.77)
**OV**	**392**	**0.01**	**1.4 (1.09 – 1.8)**
PAAD	174	0.41	1.22 (0.76 – 1.94)
PRAD	480	0.75	1.36 (0.21 – 8.92)
READ	153	0.55	1.32 (0.53 – 3.3)
SKCM	73	0.70	0.83 (0.33 – 2.13)
STAD	378	0.83	1.04 (0.76 – 1.41)
THCA	490	0.73	0.81 (0.25 – 2.63)
**UCEC**	**521**	**0.01**	**0.54 (0.34 – 0.86)**
UCS	57	0.93	0.96 (0.4 – 2.32)
UVM	76	0.33	2.49 (0.39 – 15.75)

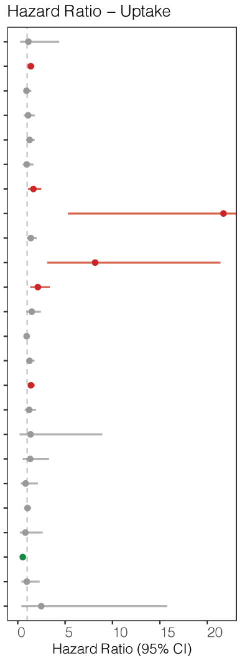

Figure S7. Comparison of hazard ratios across cancer types and cholesterol metabolism pathways. Multivariable Cox proportional hazards models were used to estimate hazard ratios and 95% confidence intervals (CI) for each cholesterol metabolism pathways): a. Biosynthesis; **b.** Catabolism; **c.** Efflux; **d.** Storage; **e.** Uptake. Models were adjusted for tumor stage and for the remaining cholesterol pathways. Histological Grade was used for GBM and LGG. Results are shown by tumor type; those highlighted in bold represent statistically significant hazard ratios. Red dots and CI indicate increased risk, whereas green indicates a protective effect.

a.

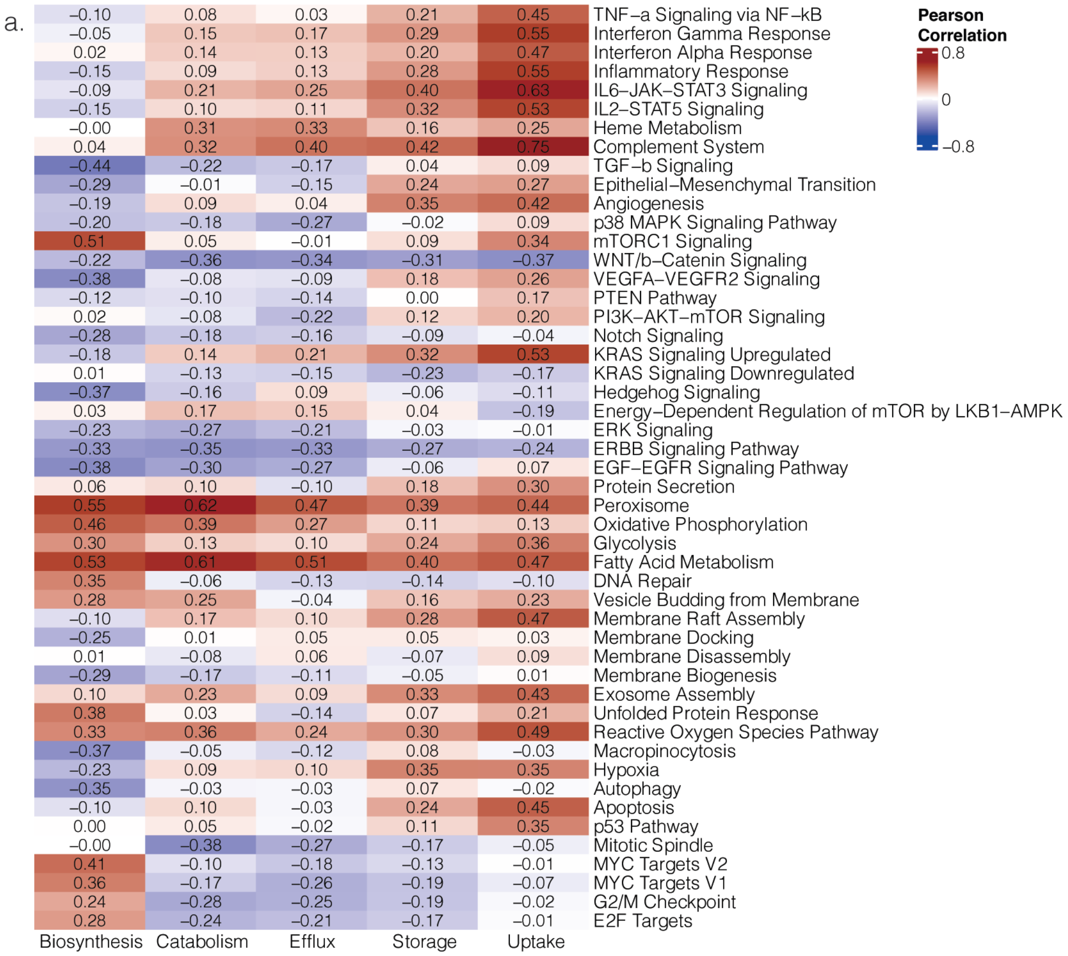

b.

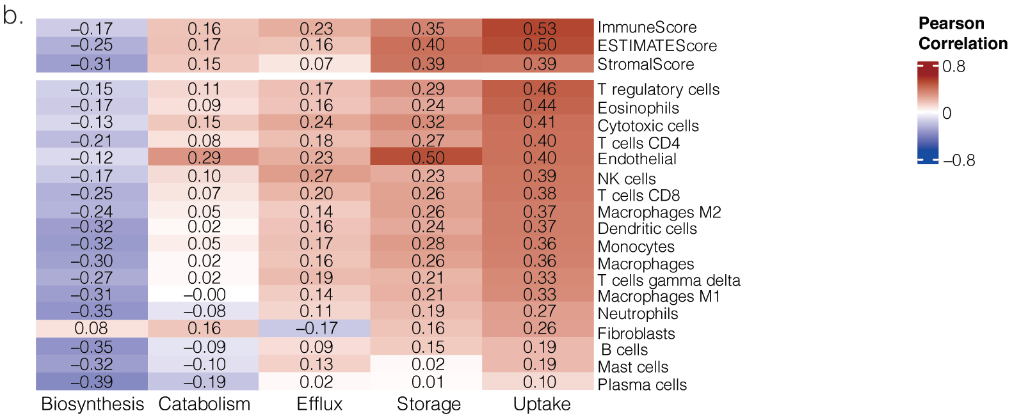

Figure S8. Correlation of tumor-associated pathways and deconvolution of immune cells population with the cholesterol-related pathways. a. Pan-cancer pearson coefficient correlation matrix between cholesterol metabolism pathways z-scores and enrichment scores of tumor-associated pathways; **b.** Pearson correlation matrix between enrichment scores of cell-specific immune signatures, obtained using ESTIMATE (top) and ConsensusTME (bottom), and cholesterol metabolism pathways scores. Cells are sorted according to the strength of their Pearson correlation with uptake, in descending order.

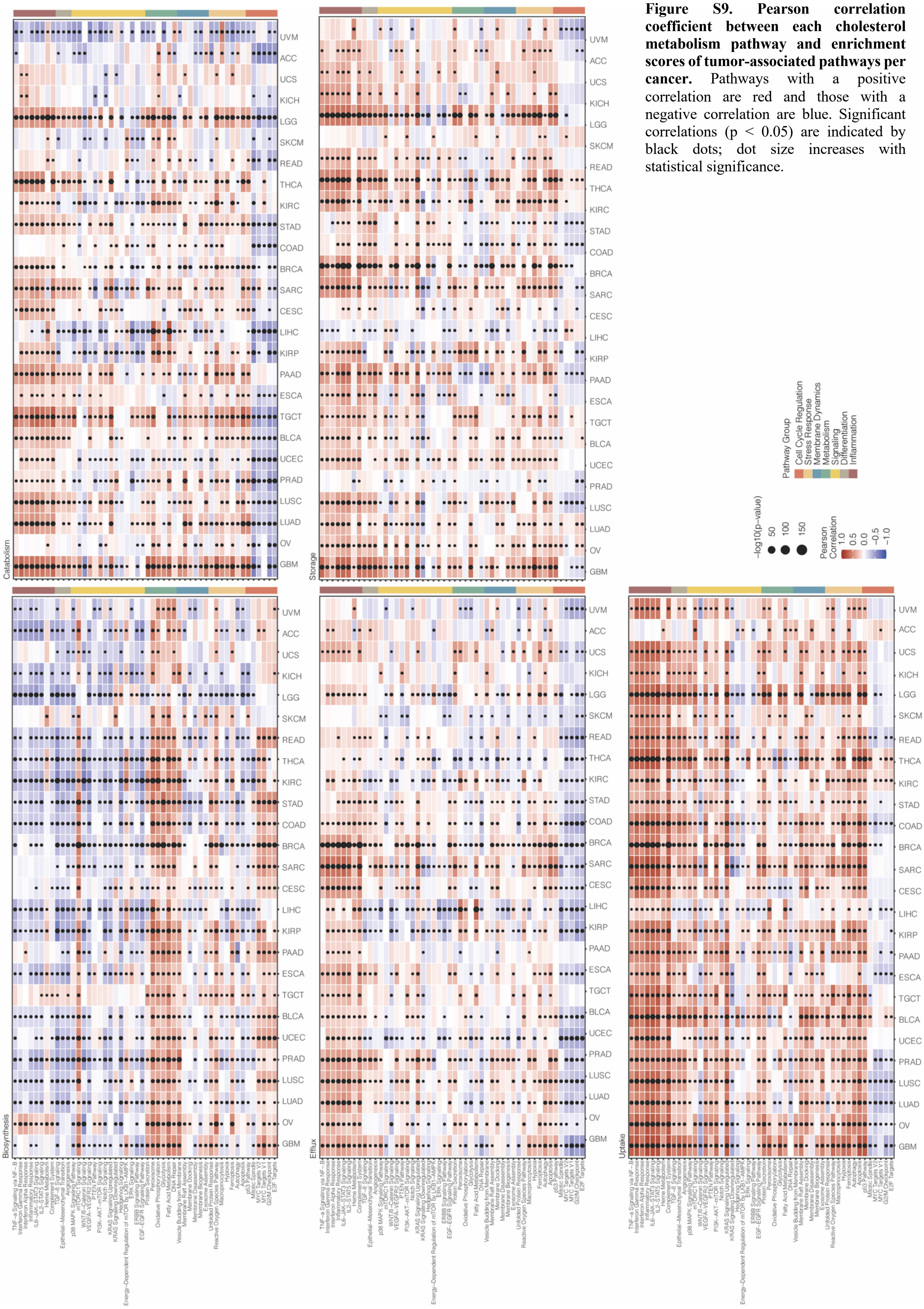

Figure S9. Pearson correlation coefficient between each cholesterol metabolism pathway and enrichment scores of tumor-associated pathways per cancer. Pathways with a positive correlation are red and those with a negative correlation are blue. Significant correlations ($p < 0.05$) are indicated by black dots; dot size increases with statistical significance.

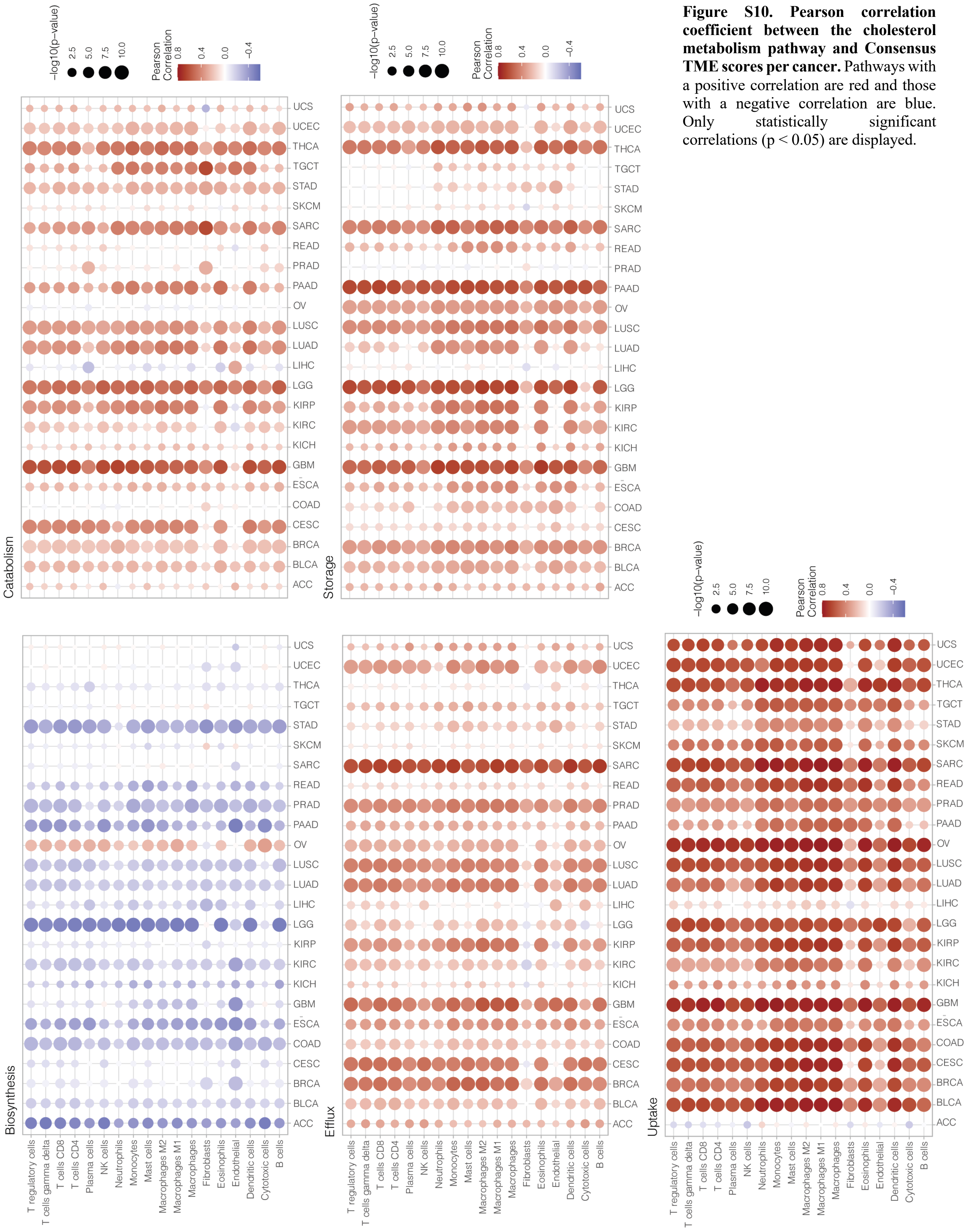

Figure S10. Pearson correlation coefficient between the cholesterol metabolism pathway and Consensus TME scores per cancer. Pathways with a positive correlation are red and those with a negative correlation are blue. Only statistically significant correlations ($p < 0.05$) are displayed.

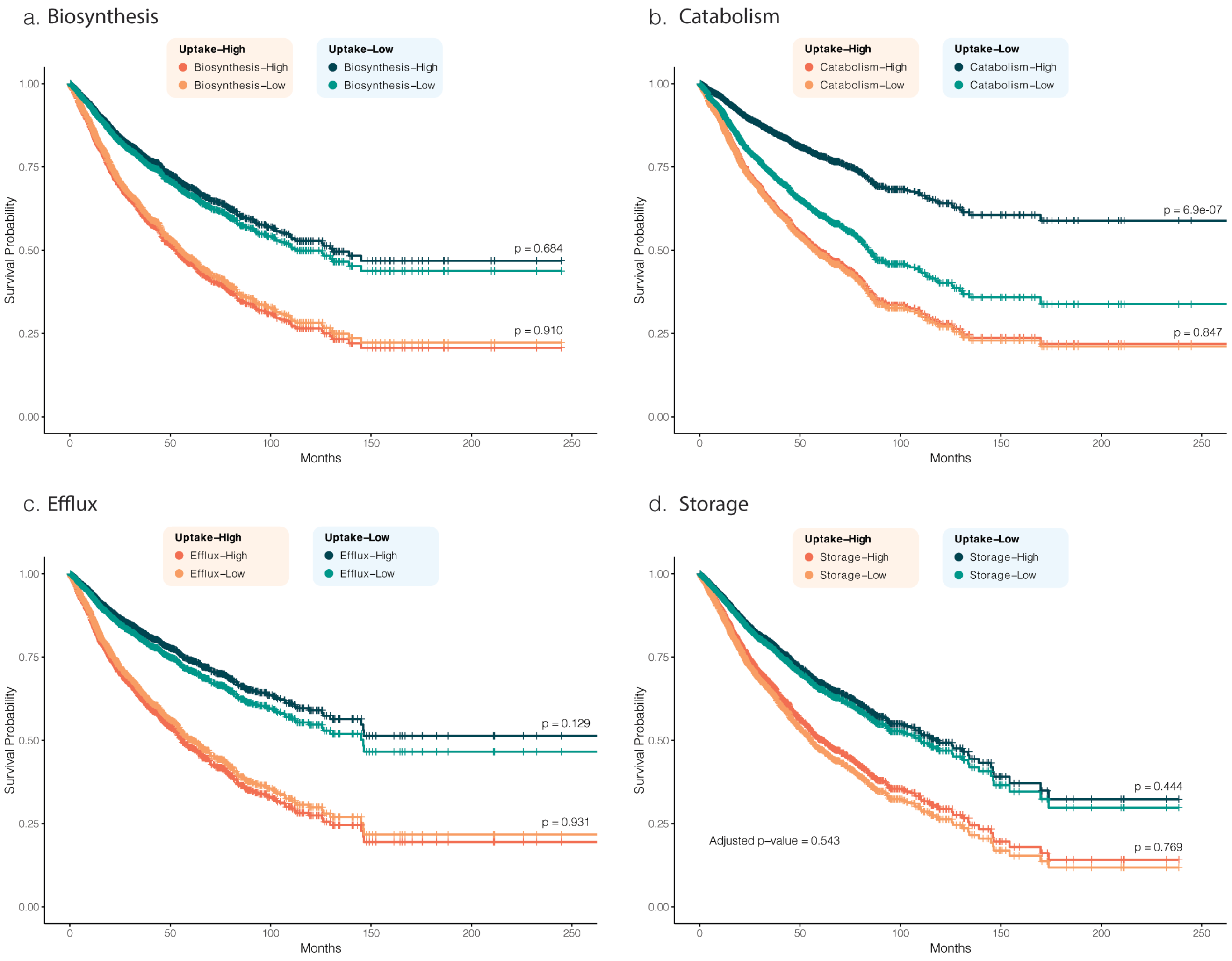

Figure S11. Cholesterol-related pathways coordinated effects with cholesterol-uptake on survival. Survival analysis was performed by combining the cholesterol uptake groups with the other cholesterol-related pathway groups. Curves show overall survival differences among the resulting patient subgroups. P-values were calculated using the Wald test. Models were adjusted for the remaining cholesterol pathways.

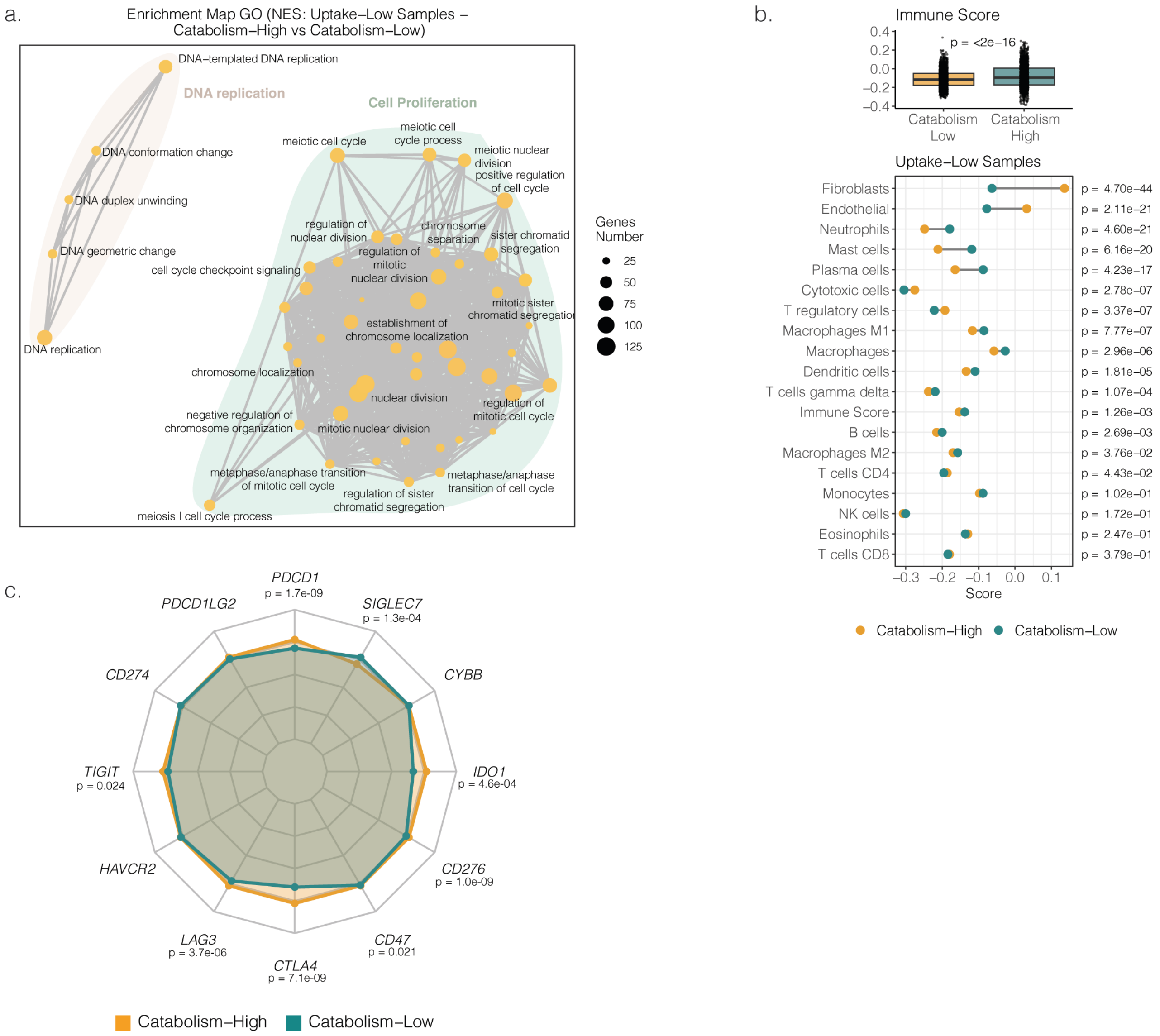

Figure S12. Differences in immune contexture across groups defined by cholesterol uptake-low and cholesterol catabolism-low or -high. **a.** Enrichment map of gene set enrichment analysis (GSEA) of uptake-low samples catabolism-low versus catabolism-high samples. Node size is related to the number of components identified within a gene set. GSEA terms associated with upregulated in the catabolism-high group are colored in orange and grouped into nodes with associated terms; **b.** Comparison of the Consensus TME immune cell populations, between the catabolism-high and catabolism-low samples; **c.** Expression of the immune supressor genes in both catabolism-high and catabolism-low groups; *p* denotes the two-sided *p*-value obtained from either a Student's *t*-test or a Mann–Whitney *U* test, depending on data distribution, which was assessed using the Shapiro–Wilk test.

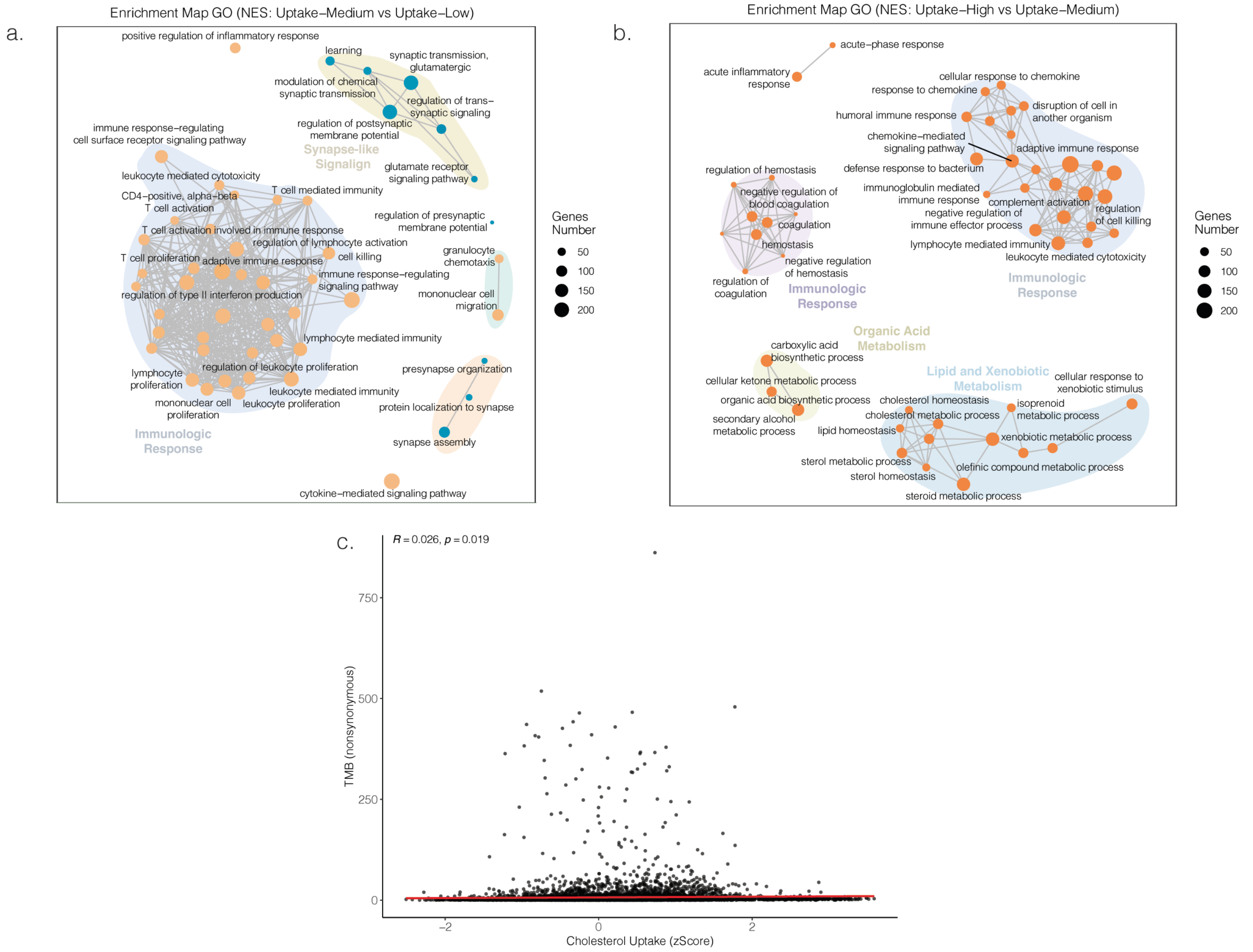

Figure S13. Enrichment map of gene set enrichment analysis (GSEA) of: a. Uptake-medium versus uptake-low versus; **b.** Uptake-high versus uptake-low samples. Node size is related to the number of components identified within a gene set. GSEA terms associated with upregulated in the uptake-high group are coloured in orange and grouped into nodes with associated terms; **c.** Pearson correlation between the tumor mutational burden (TMB) and cholesterol uptake z-score.

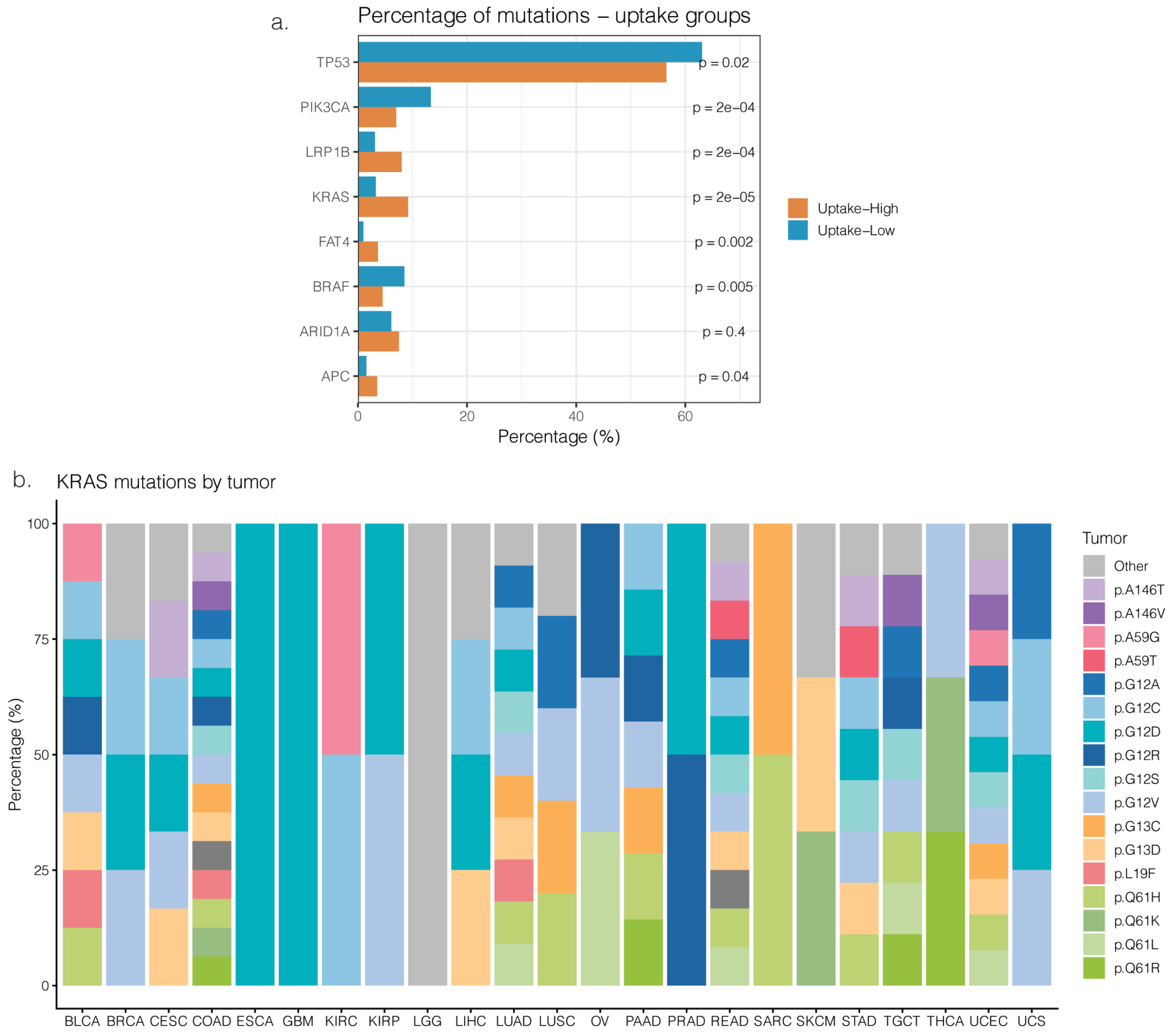

Figure S14. KRAS mutations characterization. a. Percentage of mutations in the ten most frequently mutated cancer genes, comparing high and low uptake groups; **b.** KRAS mutation distribution within each cancer type.

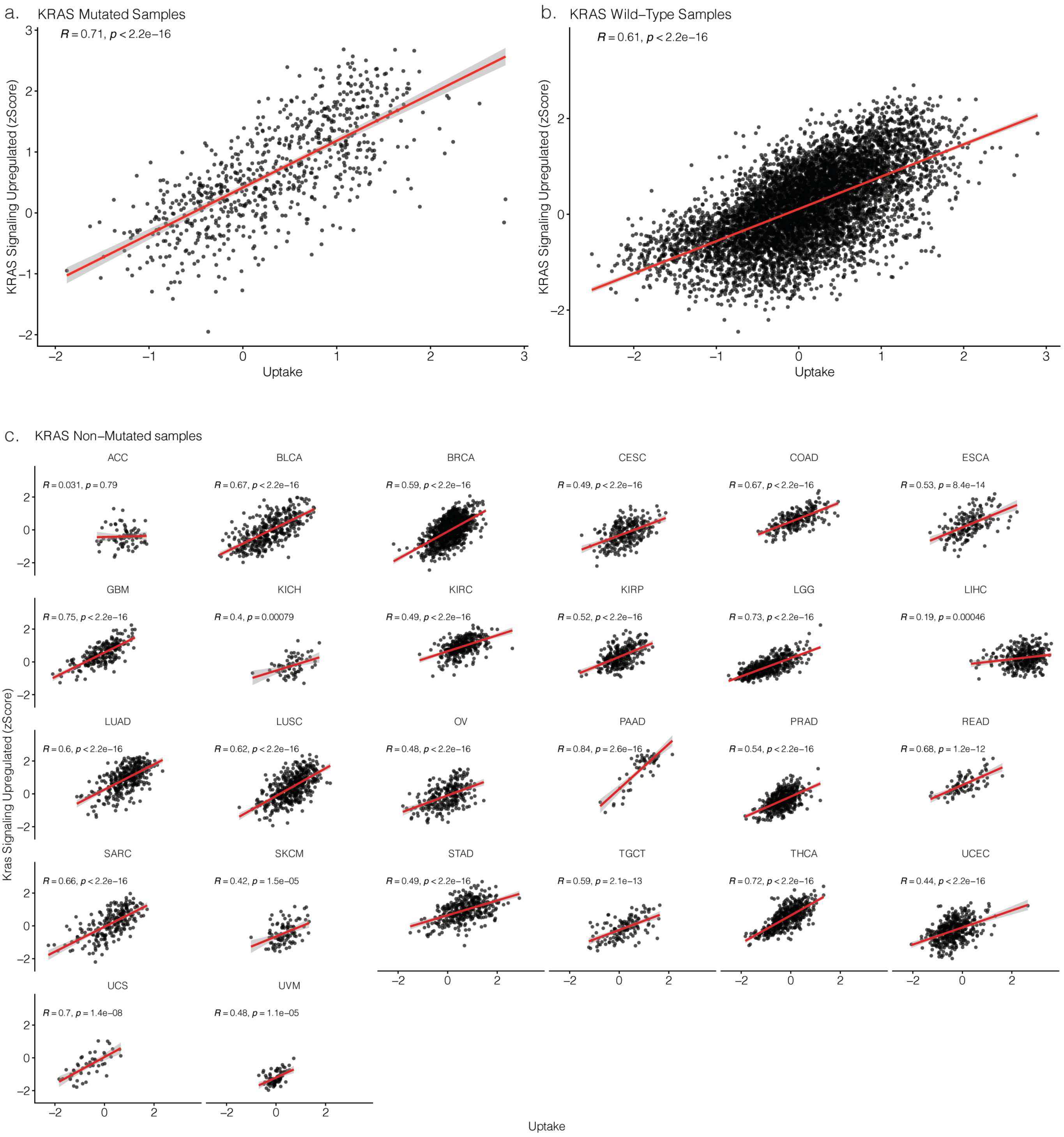

Figure S15. Association between KRAS signaling and cholesterol uptake. a. Pearson correlation of the KRAS signaling enrichment score with uptake score in the: **a.** *KRAS* mutant samples; **b.** *KRAS* wild-type samples - LIHC samples were excluded from *KRAS* wild-type due to a divergent trend; **c.** Pearson correlation between KRAS signaling enrichment score and uptake score of the *KRAS* wild-type samples for each TCGA project.
